# Examining commonly used equations for estimating the glomerular filtration rate (GFR) in a healthy cohort of children and adolescents

**DOI:** 10.1186/s12882-026-05219-y

**Published:** 2026-07-25

**Authors:** Luise Trenkmann, Niels Ziegelasch, Katalin Dittrich, Anja Willenberg, Wieland Kiess, Mandy Vogel

**Affiliations:** 1 LIFE Child Study, University Hospital for Children and Adolescents, Medical Faculty, Leipzig University, Philipp-Rosenthal-Strasse 27, 04103 Leipzig, Germany; 2 Center of Paediatric Research (CPL), University of Leipzig, 04103 Leipzig, Germany; 3 Institute for Laboratory Medicine, Clinical Chemistry and Molecular Diagnostics (ILM), University Hospital Leipzig, 04103 Leipzig, Germany; 4 Hospital for Children and Adolescents, University of Leipzig, Liebigstraße 20a, 04103 Leipzig, Germany

**Keywords:** eGFR, Kidney, Cystatin C, Creatinine, Children, Pediatric

## Abstract

**Background:**

In this study, we compared established equations for estimating the glomerular filtration rate (GFR) in a cohort of apparently healthy children and adolescents and aimed to evaluate the equations’ validity.

**Methods:**

Blood samples and anthropometric data from 4,776 apparently healthy participants (0.25–21 years) were analyzed. The glomerular filtration rate was estimated (eGFR) using the revised Schwartz Bedside (2009), the Cystatin C- and Serum Creatinine- based Schwartz equation, the Chronic Kidney Disease in Children (CKiD) equation, the 3 versions of the U25 (U25cys, U25scr, U25ave), the Serum Creatinine version, as well as the Cystatin-C based equation from the European Kidney Function Consortium (EKFCcr, EKFCcys), the CAPA equation, and the Cystatin C-Creatinine-based Chronic Kidney Disease Epidemiology Collaboration (CKD-EPI). The resulting eGFR distributions were compared, and the percentages of eGFR values within the expected physiological eGFR range (90–120 ml/min/1.73 m²) were calculated stratified by age and sex. Subsequently, age ranges with implausible distributions were identified.

**Results:**

EKFCcr, CKiD, Schwartz, and U25scr yielded the highest proportion of eGFRs within the expected range and showed consistent values across age groups without large jumps. Bland-Altman analysis indicated extremely low biases between Schwartz and EKFCcr and very high biases especially between CKD-EPI, CAPA and Bedside in reference to the other equations. Males showed higher eGFRs than females.

**Conclusions:**

We found that the EKFC equations, Schwartz, and U25 resulted in eGFR distributions within the expected physiological range for an apparently healthy pediatric cohort. Estimates from simpler equations such as Bedside and CAPA were more prone to implausible results.

**Trial registration:**

Clinical trial number NCT02550236 (clinicaltrials.gov, date of registration: 2014-12-15).

**Supplementary Information:**

The online version contains supplementary material available at 10.1186/s12882-026-05219-y.

## Background

The glomerular filtration rate (GFR) is a key marker of kidney function and in pediatrics. Accurate GFR estimation is essential not only for staging kidney function but also for mitigating nephrotoxicity risks associated with medications that rely on renal elimination.

In neonates and children, kidney function and GFR undergo significant developmental changes. During the fetal period, the placenta primarily handles the elimination of metabolic waste. Nephrogenesis, which involves growth and proliferation, is completed by the 36th week of gestation. Postnatally, GFR averages approximately 20 ml/min/1.73 m² in full-term infants [1]. Within the first 2 weeks of life, GFR increases rapidly, reaching adult levels by around 2 years of age [2, 3].

According to the 2024 KDIGO guidelines, the lower cut-off for the GFR is set at 90 ml/min/1.73 m² [4] to account for possible further deterioration during adolescence [5]. Pottel et al. proposed a slightly lower threshold of 75 ml/min/1.73 m² [6] as the lower limit for children older than 2 years, with an upper normal limit of 135 ml/min/1.73 m² [7].

Inulin is an excellent marker since the polysaccharide undergoes strict renal elimination without any tubular secretion or non-renal excretion [8]. However, this method is impractical for pediatric patients due to the challenges of precisely timed urine collection, often requiring urinary catheterization, which carries a risk of infection [9, 10].

Estimation equations have become the most widely employed tools for assessing GFR. The equations rely on endogenous markers such as serum creatinine (SCr), Cystatin C (CysC), or blood urea nitrogen, combined with biometric factors such as height, age, or sex. However, many of these equations have been derived from cohorts with kidney impairments, which can introduce biases when applied to apparently healthy children. Studies have suggested that some equations may underestimate GFR in this population [11, 12]. The Schwartz Bedside equation, which has been validated in non-CKD populations [13], has also faced scrutiny regarding its application in apparently healthy children [14]. The reliability of the U25 equation is also still debated [14]. For example, the 2024 KDIGO Guidelines continue to endorse equations using SCr as validated tools for estimating GFR in individuals aged 1–25 years [4].

In this study, we aimed to compare the performances of various estimation equations in a apparently healthy pediatric cohort and evaluate how well these estimates align with the expected normal GFR range.

## Methods

### Study population

LIFE Child is a pediatric cohort study conducted in the city of Leipzig, Germany. LIFE Child examines children’s normal development and health from pregnancy to early adulthood. It also studies the etiology of lifestyle diseases, such as obesity. The ages of the recruited subjects range from 24 weeks of gestation up to 16 years and were invited for annual follow-up assessments [15].

Our study sample is a subset of the LIFE Child Study, including all participants between 0.25 and 21 years with the necessary laboratory assessment (Table 1). Participants with signs of infections, fever, diabetes, or chronic kidney conditions were excluded. The health status, as well as the prior diagnosis of the study subjects, were ascertained through questionnaires, reports of medical history and check-ups. Further information regarding participants health status can be found in Additional File S2.

**Table 1:** Description of age (years), height (SDS), weight (SDS), cystatin C (CysC) median, Serum Creatinine (SCr) median LIFE Child cohort sorted in age interval

Characteristic	0–2		2–5		5–10		10–18		18+	
	female^1^ [1836]	male^1^ [2017]	female^1^ [1177]	male^1^ [1294]	female^1^ [2220]	male^1^ [2483]	female^1^ [3110]	male^1^ [3337]	female^1^ [190]	male^1^ [154]
Age (years)	0.72 (0.51)	0.74 (0.53)	3.50 (0.90)	3.50 (0.90)	7.52 (1.45)	7.57 (1.46)	13.55 (2.17)	13.38 (2.12)	18.85 (0.68)	18.88 (0.67)
Height group										
>10th Percentile	142 (7.7%)	121 (6.0%)	153 (13%)	126 (9.7%)	214 (9.6%)	190 (7.7%)	217 (7.0%)	216 (6.5%)	17 (8.9%)	20 (13%)
10th-90th Percentile	1,484 (81%)	1,614 (80%)	956 (81%)	1,081 (84%)	1,761 (79%)	1,987 (80%)	2,469 (79%)	2,604 (78%)	155 (82%)	121 (79%)
>90th Percentile	210 (11%)	282 (14%)	68 (5.8%)	87 (6.7%)	245 (11%)	306 (12%)	424 (14%)	517 (15%)	18 (9.5%)	13 (8.4%)
Height SDS	0.13 (0.99)	0.21 (0.98)	-0.23 (0.93)	-0.13 (0.94)	0.04 (1.01)	0.13 (0.98)	0.16 (0.99)	0.28 (1.00)	-0.08 (0.98)	-0.12 (1.08)
Weight group										
UW	160 (8.7%)	214 (11%)	38 (3.2%)	70 (5.4%)	160 (7.2%)	200 (8.1%)	249 (8.0%)	300 (9.0%)	22 (12%)	18 (12%)
NW	1,504 (82%)	1,623 (80%)	1,065 (90%)	1,127 (87%)	1,821 (82%)	2,037 (82%)	2,215 (71%)	2,399 (72%)	125 (66%)	112 (73%)
OW	109 (5.9%)	123 (6.1%)	56 (4.8%)	73 (5.6%)	96 (4.3%)	100 (4.0%)	257 (8.3%)	274 (8.2%)	16 (8.4%)	17 (11%)
OB	63 (3.4%)	57 (2.8%)	18 (1.5%)	24 (1.9%)	143 (6.4%)	146 (5.9%)	389 (13%)	364 (11%)	27 (14%)	7 (4.5%)
BMI SDS	0.01 (0.98)	0.01 (1.03)	0.09 (0.82)	0.12 (0.84)	-0.01 (1.04)	-0.06 (1.00)	0.31 (1.24)	0.20 (1.18)	0.38 (1.41)	0.11 (1.25)
Serum Creatinine (mg/dl)	0.25 (0.05)	0.25 (0.05)	0.32 (0.07)	0.33 (0.06)	0.48 (0.08)	0.48 (0.08)	0.65 (0.11)	0.69 (0.15)	0.76 (0.10)	0.94 (0.12)
Cystatin C (mg/l)	0.95 (0.13)	0.95 (0.14)	0.84 (0.10)	0.86 (0.11)	0.88 (0.10)	0.87 (0.10)	0.87 (0.12)	0.94 (0.12)	0.81 (0.10)	0.90 (0.09)

^*1*^ Mean (SD); n (%); SDS (Standard deviation score), UW - underweight, NW - normal weight, OW - overweight, OB - obese

This table shows a description of age (years), height (SDS), weight (SDS), cystatin C (CysC) median, Serum Creatinine (SCr) median LIFE Child cohort stratified by age.There may be multiple observations of participants that belong to different age intervals. BMI groups: underweight < 10th percentile, overweight > 90th percentile, obese > 97th percentile, n number of observations, SD standard deviation, BMI body mass index

### Measures

Height and weight were determined following standardized procedures by trained study personnel. BMI was calculated and subsequently transformed into age- and sex-adjusted standard deviation scores following the guidelines of the German Obesity Society and the German Society of Pediatrics and Adolescent Medicine (DGKJ). Using the same guidelines, weight groups were defined as underweight (BMI-SDS < -1.28), normal weight (-1.28 < BMI-SDS < 1.28), overweight (1.28 < BMI-SDS < 1.88), and obese (BMI-SDS > 1.881) [16]. Height groups were defined as < 10th Percentile (Height-SDS < -1.28), 10th Percentile to 90th Percentile (-1.28 < Height-SDS < 1.28), and > 90th Percentile (Height-SDS > 1.881) [16].

We categorized values as inside/outside the physiological range of eGFRs as 90–120 ml/min/1.73m^2^, following the KDIGO guideline [4] for all age groups except those under 2 years old, because they are assumed to have a deviating physiological range.

Age groups were defined to account for increasing eGFR in infancy [2, 3] and potential changes during puberty [17]: infants (< 2 years), preschool children (2–5 years), primary school children (> 5–10 years), adolescents (> 10–18 years), and adults (> 18 years).

The venous blood samples, using serum monovettes (Sarstedt AG&Co, Nümbrecht, Germany), were analyzed by the Institute for Laboratory Medicine, Clinical Chemistry and Molecular Diagnostics (ILM) at the University Hospital Leipzig on an automated laboratory analyzer Cobas 8000 (Roche Diagnostics, Mannheim Germany) according to the manufacturer’s protocol.

Serum creatinine (SCr) was measured with an enzymatic assay (Roche Diagnostics). Blood Urea Nitrogen (BUN) was performed with a kinetic test using urease and glutamate dehydrogenase (GLDH) (Roche Diagnostics). Cystatin C (CysC) was determined using the turbidimetric immunoassay (PETIA) Tina-quant^®^ Cystatin C (Roche Diagnostics, measurement range: 0.4–8.0 mg/l), standardized against a Roche in-house reference preparation of recombinant human CysC. Since 2015 the measurement was done with the second-generation Tina-quant^®^ Cystatin C-assay (Roche Diagnostics, primary measurement range: 0.4–6.8 mg/l), which has been standardized against the international reference material ERM-DA471/IFCC [18]. Both methods showed good conformity and no relevant bias [17].

### Statistics

eGFR was calculated by applying the different equations and functions from kidney.epi package [19]. We calculated eGFRs within the 90–120 ml/min/1.73m^2^ range as well as within the 75–135 ml/min/1.73m^2^ range for all age groups. Associations between each of the eGFR (Schwartz, CKiD, U25ave) measures as outcomes and sex, weight group, or height group as predictors were estimated using hierarchical linear models, and we adjusted for multiple measurements per child by adding the subject to the model as a random effect. Furthermore, we used the Bland-Altman analyses to compare the equations. No correction for multiple comparisons was applied. The statistical tests performed are not independent: they address the same underlying question across related subgroups and equations. Further, the dependency structure between the tests cannot be adequately captured by standard correction procedures. Readers are therefore encouraged to interpret individual findings in the context of the overall pattern of results rather than relying on any single p-value in isolation. Furthermore, as the primary aim of this study is to detect systematic biases in equation performance, Type II error (failure to detect a true bias) is of greater concern than Type I error; correction for multiple comparisons, which reduces statistical power, would therefore be counterproductive in this context. All statistical analyses and visualizations were implemented with the R software (R 4.3.2, R Core Team).

### Equations

We included the most commonly used eGFR equations (Table 2). The revised Schwartz Bedside (2009) is one of the simpler equations, including only the variables height and serum creatinine [20]. We also included the full CysC- and SCr-based Schwartz equation, as well as the CKiD equation [20, 21]. The three variations of U25 use CysC (U25cys) or SCr (U25scr), and the averaged value (U25ave) [22]. To contrast the SCr-based Schwartz Bedside calculation, we added the CAPA equation, which is also a simpler calculation based only on CysC and age [23]. Since our cohort also included adolescents and a few young adults and young adults, and given that the CysC-SCr-based CKD-EPI [24] is the most widely used adult eGFR equation internationally, it was included as a deliberate comparative reference. Its inclusion serves two purposes: first, to assess its behavior in older adolescents approaching the transition to adult care; and second, to provide an internal benchmark illustrating the limits of extrapolating adult-derived equations to a paediatric population. Furthermore, we used the European Kidney Function Consortium (EKFC) equations, which are modified versions of the FAS-equation, using CysC(EKFCcys) [25] and SCr(EKFCcr) [26] respectively. The complete equations are listed in Additional File S1.

**Table 2:** Overview of used eGFR equations

Equation	Age	Sex	Height	Serum Creatinine	BUN	Cystatin C	Age range
Schwartz Bedside (2009) [20]			x	x			1–18
Schwartz (2009) [20]		x	x	x	x	x	1–19
CKiD (2012) [21]		x	x	x	x	x	1–18
CAPA (2014) [23]	x	x				x	> 1
U25cys (2021) [21]	x	x				x	1–25
U25scr (2021) [22]	x	x	x	x			1–25
U25ave (2021) [24]	x	x		x		x	1–25
CKD-EPI (2021) [24]	x	x		x		x	> 18
EKFCcys (2021) [25]	x					x	18–100
EKFCcr (2023) [26]	x			x			2-100

This table shows which laboratory or anthropometric measures are used to calculate the GFR for each equation, respectively, as well as the intended age range

## Results

**Fig. 1:**
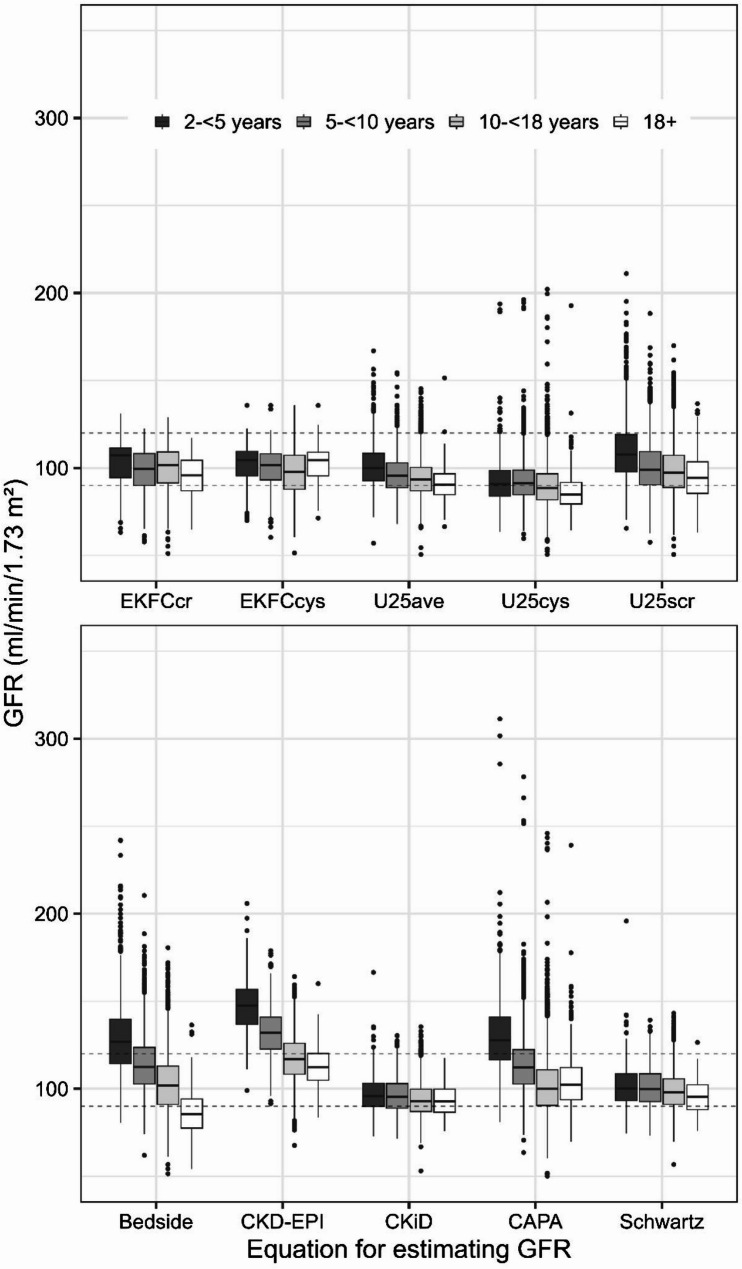
Boxplot of eGFRs grouped by age and equation. This figure contains two panel boxplots showing GFR estimates (ml/min/1.73 m²) from different GFR estimating equations, stratified by age group. The dotted line represents the upper and lower cutoffs for the GFR in apparently healthy pediatric older than 2 years of age (90-120 ml/min/1.73m²)

The EKFCcys, EKFCcr, CKiD, and Schwartz showed consistent GFR estimates across all age groups, without large jumps between adjacent age groups, with means clustering between 88 and 106 ml/min/1.73 m² and relatively narrow interquartile ranges. In contrast, U25-based equations, particularly U25scr, produced higher GFR values in 2-to-5-year-olds, with mean values between 92 and 113 ml/min/1.73 m². The U25cys estimated lower GFR values, especially considering the adult population with mean values below 90 ml/min/1.73m^2^. The Bedside and CAPA equations tended to estimate very high GFR in children under 10, with median values exceeding 120 ml/min/1.73 m² and extensive spread. Even higher values were reached when we used CKD-EPI, with the median well above and the first quartile near the upper reference limit for 2-to-10-year-old children (Table 3; Fig. 1).

Only for U25scr and both EKFC equations, more than 50% of the eGFR fell within the reference range across all age groups (Table 4). The EKFCcys showed the highest percentages within the range (59–95%). CKiD, Schwartz, and U25ave showed similar results, except for the adult population. The remaining equations showed marked discontinuities between age groups as well as very low percentages within the reference range for several age groups (Table 4). The results when the 90–135 ml/min/1.73 m² cutoff were applied are shown in Additional File S3.

**Table 3:**
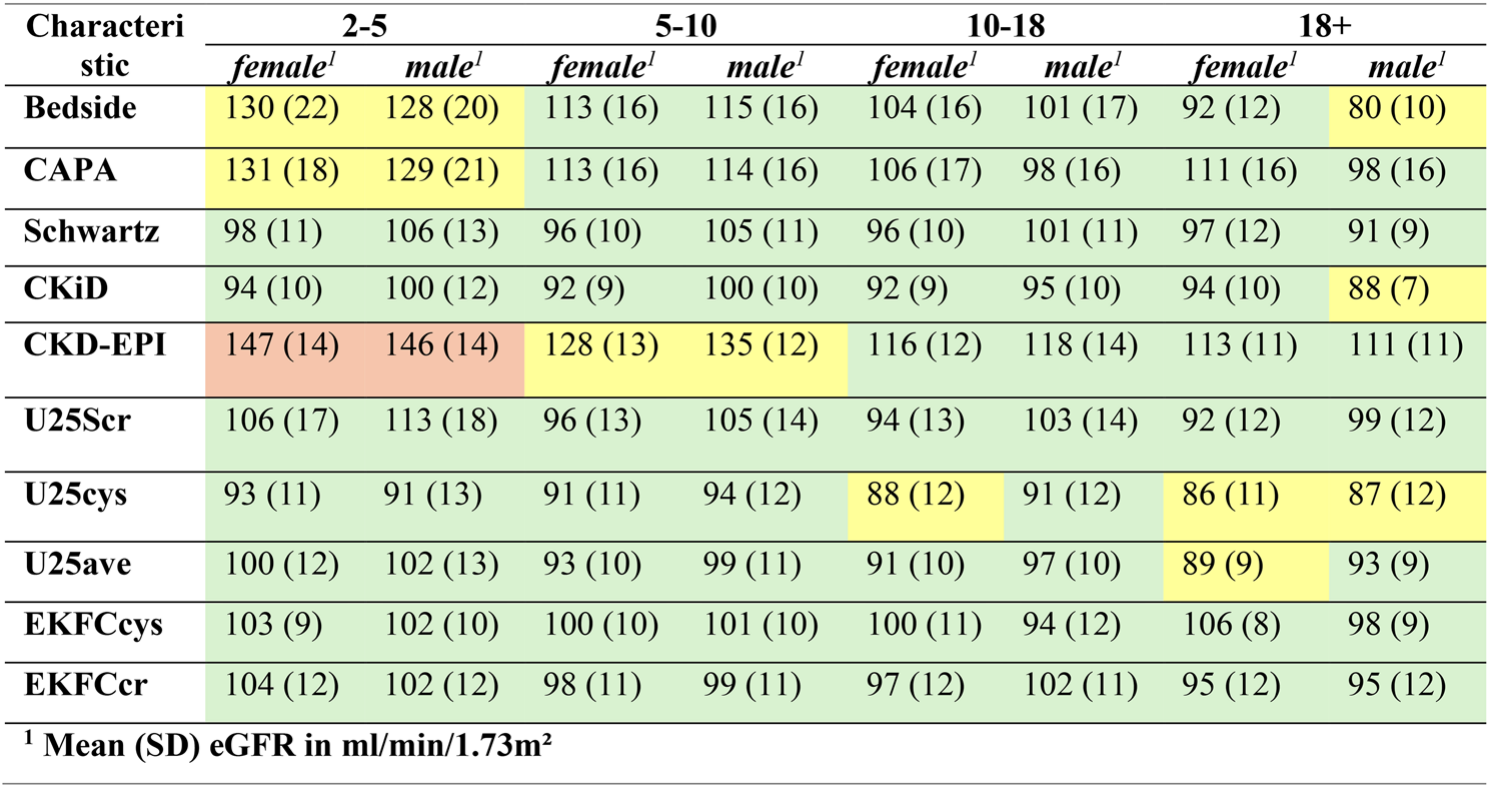
Mean and SD of eGFR for each equation grouped by age (in years)

This table shows the mean and standard deviation (SD) of the eGFRs for each equation. For visual clarity, eGFR values are color-coded as follows: green (90–120 ml/min/1.73m², within the normal range), yellow (±15 ml/min/1.73m² deviation from the expected range), and red (≥15 ml/min/1.73m² deviation), indicating normal, mildly altered, and severely altered values, respectively. There may be multiple observations of participants that belong to different age intervals

**Table 4:**
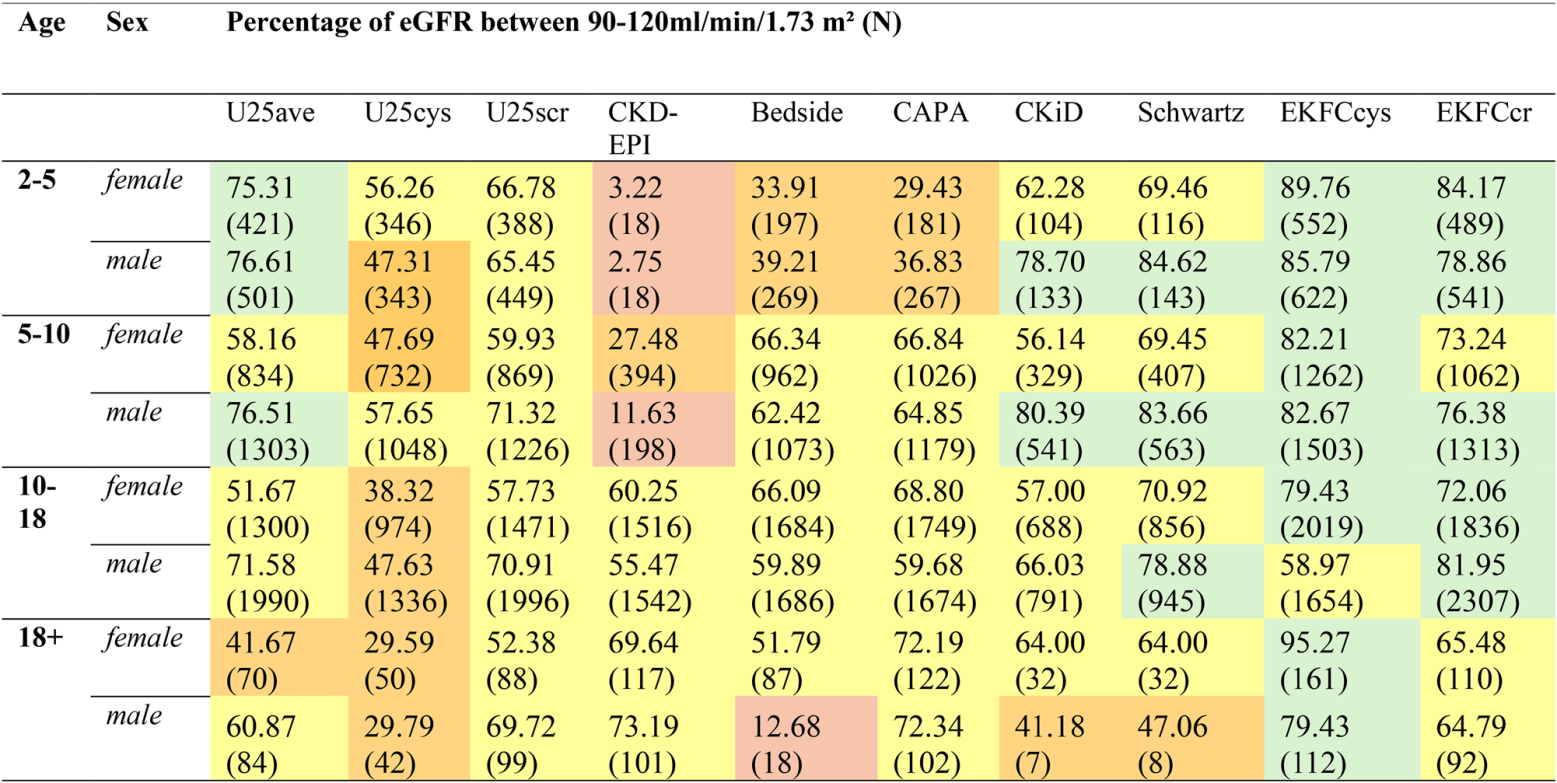
Percentage of estimated GFR between 90-120ml/min/1.73m² (N) grouped by age (years)

The table shows the percentage of results within the normal eGFR-range, the number of measurements is stated in parentheses. For visual clarity, percentage values are color-coded as follows: green (≥75%), yellow (≥50%), orange (≥25%) and red (<25%)

### Bland-altman analysis

**Fig. 2:**
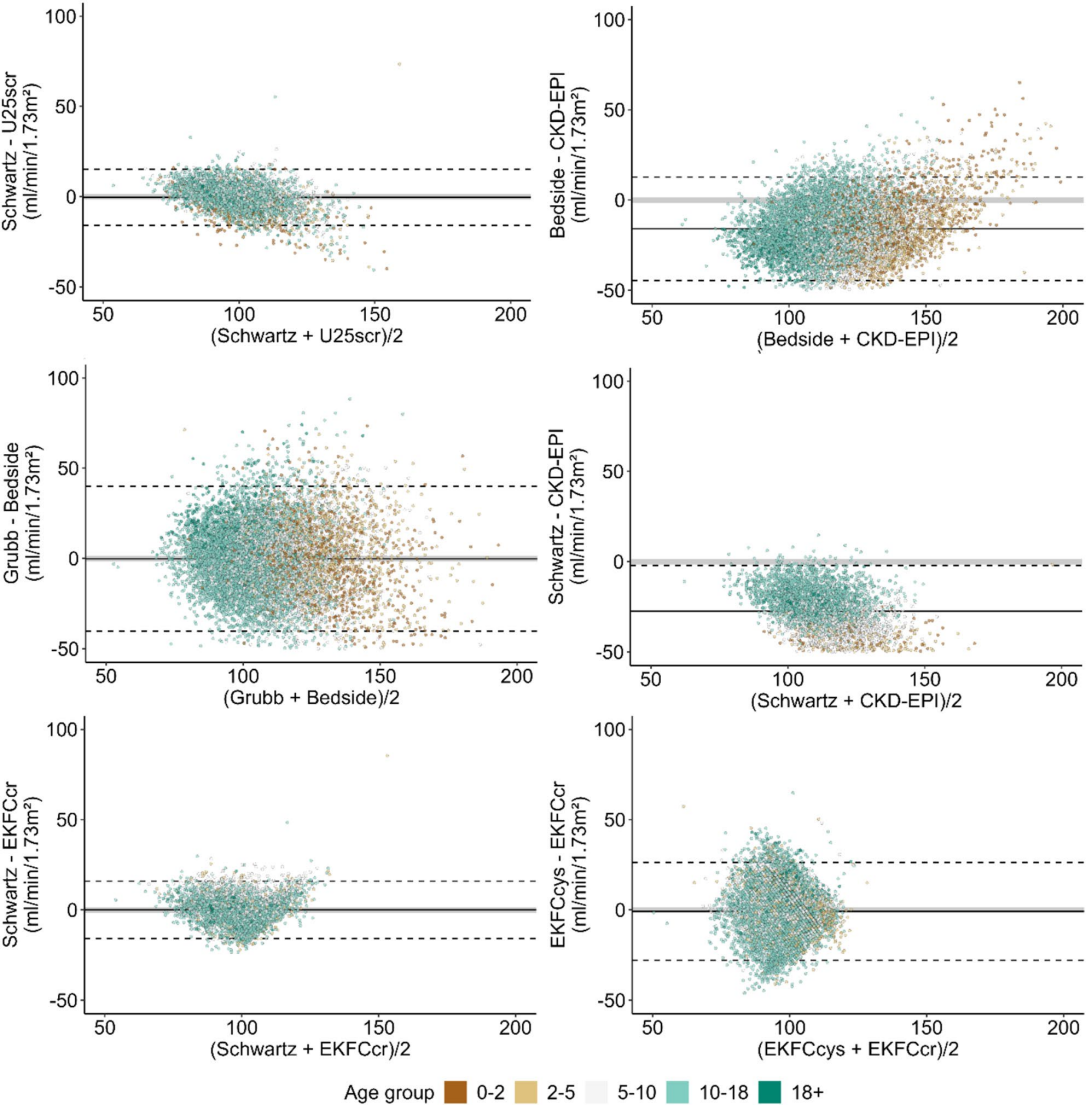
Bland-Altman-Plots plotting the difference between the equations against their averages, grouped by age. This figure contains Bland–Altman plots illustrating agreement between each estimated glomerular filtration rate (eGFR) equation and their averages across different age groups. The x-axis represents the mean of the two compared equations, and the y-axis shows their difference. Each point represents an individual observation, color-coded by age group (0–2 years, 2–5 years, 5–10 years, 10–18 years, and ≥18 years). Panels show comparisons for: U25 average, U25 cystatin C, U25 creatinine, CKD-EPI, CAPA, CKiD, Schwartz and FAS creatinine equations. The solid black horizontal lines indicates mean bias, the solid light-grey line indicates the ideal (zero bias), while the dashed lines represent ±1.96 standard deviations (limits of agreement). The plots show varying consistency between two different eGFR equations.

We found the eGFR estimated by the EKFCcr to be closest to those estimated by the Schwartz equation as a reference (bias= -0.04, proportional bias= -0.001). The bias was also very low for U25scr and Schwartz (0.35), EKFCcr and EKFCcys (-0.85). Low biases were found between the EKFC, CKiD, Schwartz, and U25scr and U25ave equations. By contrast, while CAPA and Bedside showed a very low constant and proportional bias (0.19, 0.041) in reference to each other, they result in larger biases with CKDEPI and especially with the other equations, indicating considerably different GFR estimates. Indeed, the corresponding Bland-Altman plot show considerable variation, suggesting only limited agreement between the equations (Fig. 2). All between-equation comparisons, as well as Bland-Altman plots can be found in Additional File S4 as well as Additional Files S5-13.

### Associations with sex, weight, and height group

**Fig. 3:**
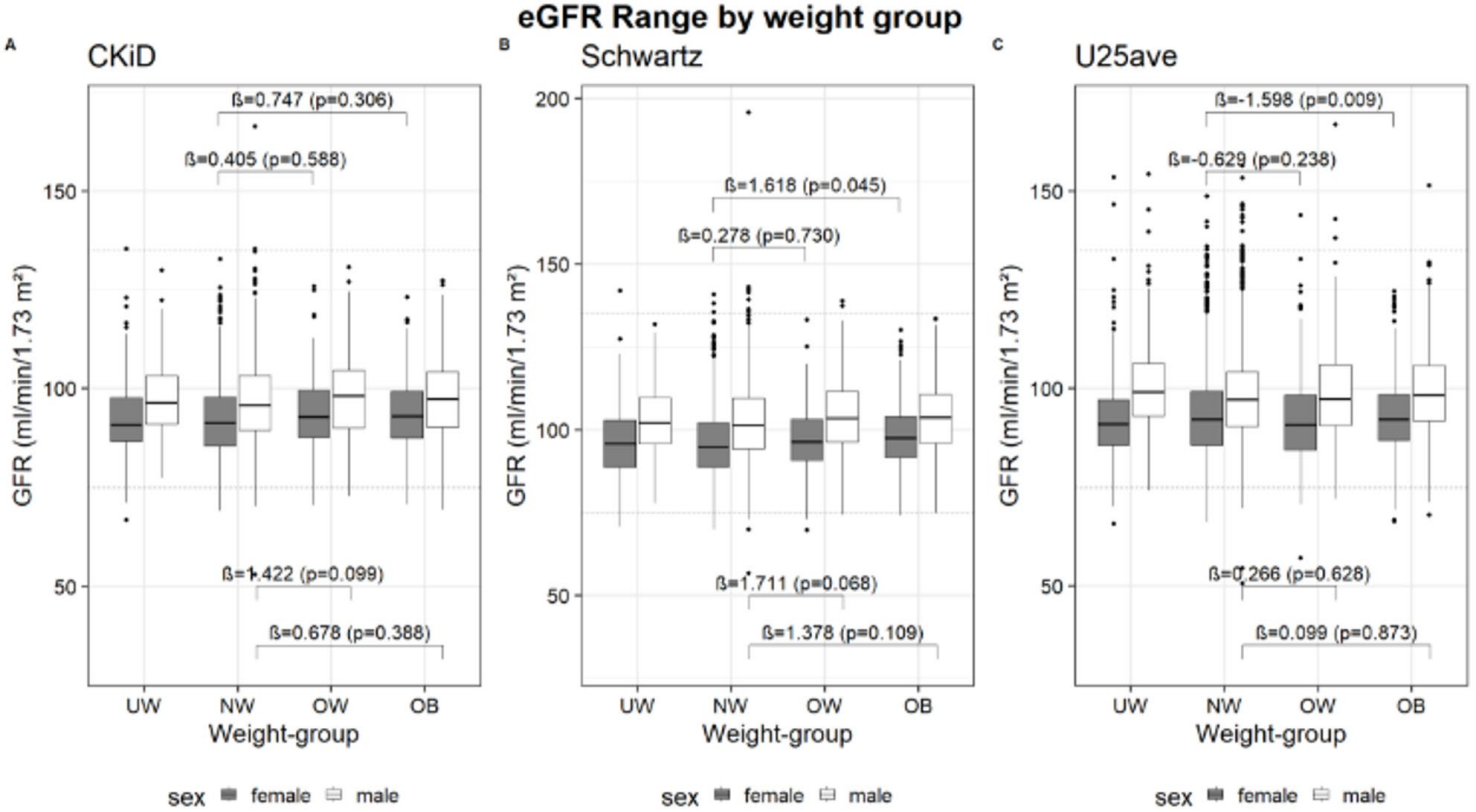
Boxplots showing the eGFR for weight, grouped by sex. This figure contains box plots showing the distribution of eGFR (ml/min/1.73 m²) across weight groups (underweight (UW), normal weight (NW), overweight (OW), and obese (OB)) stratified by sex (female, grey boxes; male, white boxes) (**A**) eGFR calculated using the CKiD equation (**B**) eGFR calculated using the Schwartz equation (**C**) eGFR calculated using the U25ave equationHorizontal dashed lines represent the 75-135 ml/min/1.73m² range.

To allow a deeper analysis of how height and weight may influence estimated kidney function, we focused on a selection of three widely used equations (Schwartz, CKiD, U25ave). For the three investigated equations eGFRs were consistently higher in males than females across the entire study population as well as within all weight groups (Fig. 3, all *p* < 0.001). For the Schwartz equation and the CKiD, we found higher eGFRs for children with overweight and obesity than for normal weight children. For Schwartz, these differences reached statistical significance only for girls with obesity. For CKiD, the comparisons yielded similar results but showed only weak effects that were not statistically significant. No consistent patterns were found for U25ave (Fig. 3).

**Fig. 4:**
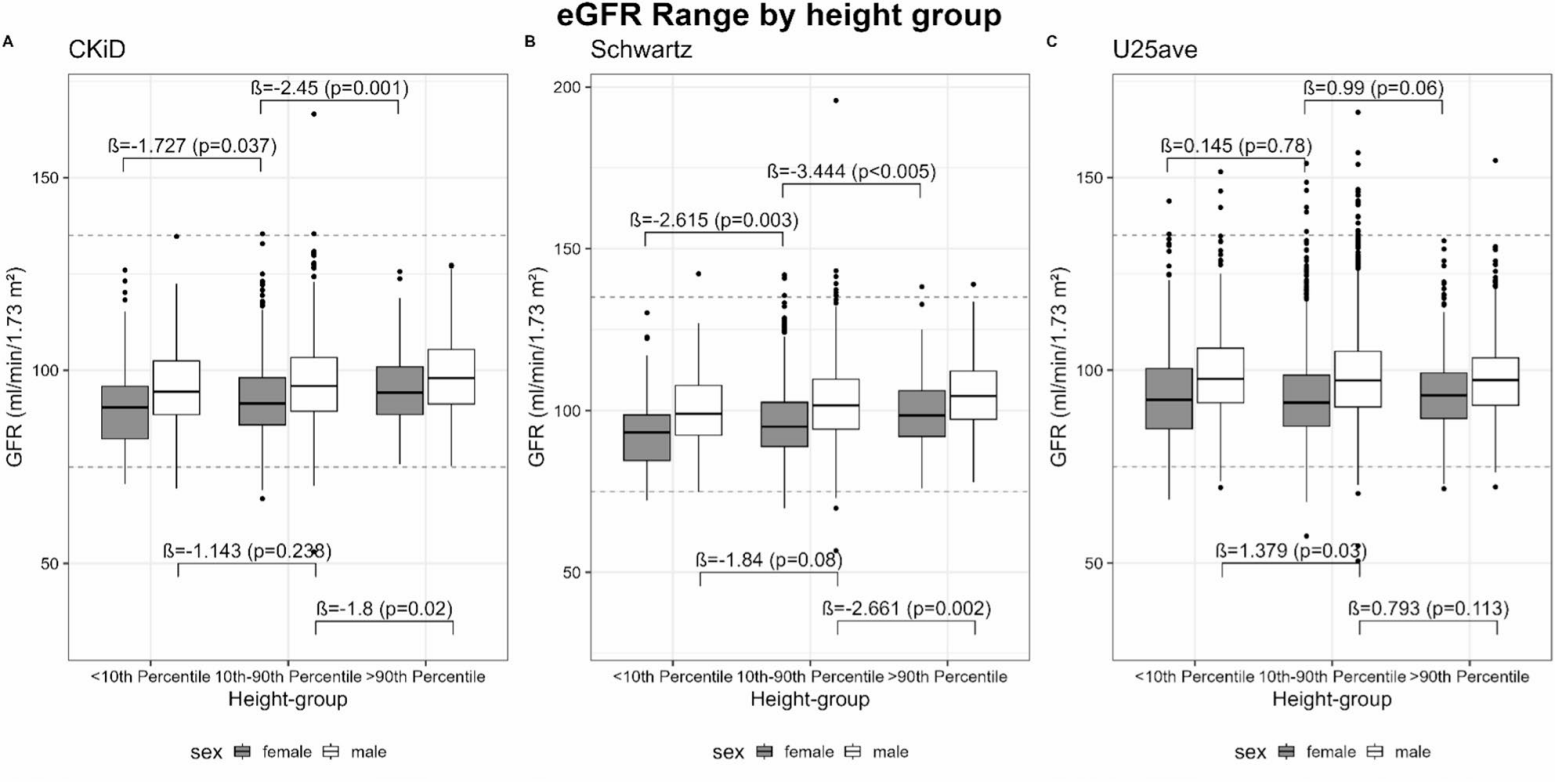
Boxplots showing the eGFR for height, grouped by sex. This figure contains box plots showing the distribution of eGFR (ml/min/1.73 m²) across height groups (10th Percentile, 10th Percentile to 90th Percentile, and > 90th Percentile) stratified by sex (female, grey boxes; male, white boxes) (**A**) eGFR calculated using the CKiD equation (**B**) eGFR calculated using the Schwartz equation (**C**) eGFR calculated using the U25ave equationHorizontal dashed lines represent the 75-135 ml/min/1.73m² range.

For Schwartz and CKiD, we found higher eGFRs in taller children for girls as well as for boys. For the U25ave, estimated eGFRs were similar across the height groups (Fig. 4).

## Discussion

Chronic kidney failure is a global health problem and a leading cause of morbidity and mortality for millions of people. Chronic kidney insufficiency often progresses silently, and by the time individuals develop significant clinical symptoms, kidney function has already been severely impaired. To implement a preventive approach, methods for assessing kidney function are relevant for all age groups [4].

Accurate eGFR estimation is crucial for assessing kidney health, adjusting medication dosages, and mitigating nephrotoxic risks associated with various pharmaceuticals. The reliable estimation of GFR is particularly important in children, as absolute values of kidney retention parameters such as serum creatinine (SCr) or cystatin C (CysC) undergo changes during phases of growth and puberty, making it difficult to assess overall kidney function accurately. Our study population was ethnically homogenous (> 98% Caucasian/white) and therefore our findings may not be generalizable to other ethnicities. The usage of race as a factor in eGFR equations has been a topic of debate [24, 26]. According to the KDIGO 2024 guidelines, eGFR equations should consider geographical region. In our study, we used equations like the EKFC, using standardized Q-values for Africa and Europe, as well as the Schwartz and CKiD, which are based on data from North American cohorts. Our Bland-Altman analyses showed strong agreement between, e.g., the North-American-based Schwartz and CKiD equations, the European-based EKFCcr, and the U25, which was based on a multiracial cohort.

Sex-related differences in kidney parameters, including kidney size and nephron number, have been described. However, differences in eGFR estimates remain a topic of debate, particularly in prepubertal and early adolescent populations, and should be interpreted cautiously. Such differences may reflect variations in body composition, muscle mass, hormonal influences on endogenous markers, and sex-specific adjustments incorporated into eGFR equations rather than true differences in measured kidney function[27–29]. Most equations tend to estimate higher GFR values in males due to these physiological differences [30]. For example, markers such as Cystatin C, while less influenced by muscle mass, still show associations with age, sex, and pubertal stage [17]. In our cohort, sex differences were evident across all equations incorporating sex as a factor, with females generally showing lower eGFR values. These findings are consistent with studies in adult populations, where women are often reported to have lower baseline kidney function [31, 32]. Still, the EKFC equations, which do not include sex, showed a very high proportion of estimates within the expected physiological range of 90–120 ml/min/1.73 m² range. Among the three versions of the U25 equation, the one including serum creatinine demonstrated slightly better consistency between age-groups. The EKFCcys showed slightly more eGFRs within the expected range than the EKFCcr. Further, we found only small biases between equations using either or both markers, which indicate that these markers perform similiarlly within our study. These findings are timely in the context of ongoing debate about the role of cystatin C in paediatric GFR estimation. While recent proposals advocate for broader incorporation of cystatin C into KDIGO guidelines for children [33], our results suggest that the performance of cystatin C-based equations is markedly dependent on the specific equation used: EKFCcys showed greater consistency across age groups, whereas CAPA showed greater instability, particularly in younger children. This underscores that the choice of equation matters as much as the choice of biomarker, and that the transition to cystatin C-based estimation in paediatric practice should be accompanied by careful evaluation of which equation is applied.

The pubertal divergence in serum creatinine (Additional File S16) observed in our cohort has direct implications for the choice of age partitioning in studies such as this one. Our data show that sex-related differences in serum creatinine are negligible through approximately age 12–13 years and accelerate markedly from age 14 onward, consistent with the pubertal increase in lean muscle mass in adolescent boys. This pattern supports the biological rationale for distinguishing a prepubertal group (up to age 12–13) from a pubertal and post-pubertal group (from age 13 onward). Importantly, the absence of meaningful sex differences in serum creatinine before age 13 suggests that sex-stratified eGFR interpretation in the prepubertal range should be approached cautiously, as apparent sex differences in eGFR in this age group may reflect equation structure rather than true physiological differences in kidney function. A supplementary analysis using biologically motivated age partitions (< 2, 2–12, 13–17, and 18–25 years) is provided, which, while not showing as strong an adherence to the 90–120 ml/min/1.73 m² range, nevertheless confirms that the main conclusions of this study are robust to the choice of age grouping. (Additional File S14-15)

A notable finding of this study is that several equations, particularly Bedside, CAPA, and CKD-EPI in the youngest age groups, produced eGFR values substantially exceeding the upper physiological reference limit of 135 mL/min/1.73 m² [7]. While the absence of a reference GFR measurement (e.g., iohexol clearance) precludes definitive assessment of absolute accuracy, there are compelling reasons to interpret such extreme values as equation artefacts rather than genuine physiological findings. Our cohort underwent a comprehensive, multi-layered health ascertainment process including paediatrician-led anamnestic interviews, structured medication screening, family medical history, and repeated longitudinal follow-up visits from infancy. In such a carefully characterised cohort, the widespread occurrence of true glomerular hyperfiltration, a pathological state associated with obesity, early diabetic nephropathy, and compensatory nephron hypertrophy, would be highly implausible. Most importantly, the same participants yielded markedly discrepant eGFR estimates depending solely on the equation applied, a pattern that cannot reflect genuine inter-individual variation in GFR and instead directly demonstrates the mathematical consequences of differences in equation structure, biomarker weighting, and derivation cohort. This cross-equation inconsistency is not merely a statistical observation. It has direct clinical implications, as a child’s classification as having normal, high-normal, or hyperfiltrating kidney function may depend entirely on which equation their clinician uses. The primary contribution of this study therefore lies in evaluating how different equations classify apparently healthy children in relation to established reference limits, rather than in bench marking absolute accuracy; a question that is clinically meaningful in its own right.

On the other hand, considering the KDIGO guideline threshold of 90 ml/min/1.73 m² for normal kidney function, a greater proportion of female participants in our cohort fell below this limit. However, we would like to emphasize that an eGFR between 75 and 90 ml/min/1.73 m², in the absence of albuminuria or hematuria, still represents a low risk for the development of end-stage kidney failure [4]. Therapeutic interventions such as adjustments of medication dosages or avoiding contrast agents are typically considered only for eGFR values below 60 ml/min/1.73 m² [34]. Thus, the methods that are commonly used to estimate GFR can be applied for both sexes in these decisions.

The ongoing pandemic of childhood obesity is expected to contribute to an increased risk of chronic kidney disease (CKD) in later life, as early-life adiposity has been linked to long-term CKD development [35, 36]. Van Dam et al. found correlations between elevated BMI and below-average values for creatinine-based eGFRs, depending on which equation they used [37]. In our cohort, we found no evidence of impaired GFR at higher weight or overweight. This result may have occurred because the overweight patients are still in the “hyperfiltration” phase, with eGFR values above 135 ml/min/1.73 m². On the other hand, our selection approach may have excluded individuals with underlying kidney impairment, potentially introducing selection bias. But nonetheless, across the three equations we investigated, higher eGFR values were consistently observed for children with overweight and obesity compared to normal weight peers, which is in line with findings from similar studies [38, 39] and supports the validity of these equations in reflecting the early hyperfiltration phenomenon. This constellation may lead to nephrosclerosis and a significant loss of eGFR in the medium term.

Adolescents and young adult populations require special consideration. The transition from pediatric to adult care often involves a shift in the equations used for eGFR, which can lead to variability in reported kidney function [40]. This transition period highlights the need for standardized approaches that can account for the physiological and developmental changes that occur during this stage of life. The CKD-EPI equation is designed for individuals 18 years of age and older; prior research has shown its limited reliability not only in children but also in young adults [41–43]. Our results align with these observations showing substantial differences in eGFR estimates across age groups and unusually high mean eGFR values in younger participants. This was anticipated: the inclusion of CKD-EPI was intentional, serving as a comparative benchmark to illustrate the risks of extrapolating adult-derived equations to paediatric populations. Reassuringly, its performance improved in participants older than 10 years, suggesting some applicability at the upper end of the paediatric age range. Further, the U25 and EKFC equations, which were partially developed to bridge this gap, yielded eGFRs within the expected range and consistent results across age groups. In the absence of measured GFR, clinicians evaluating apparently healthy children may prefer EKFC or Schwartz-based equations, while Bedside and CAPA should be interpreted cautiously in children under 10.

### Limitations

We should consider that our results are potentially biased through the equations themselves.

The mathematical formulation of several equations may influence their behavior. For instance the EKFC equations introduce a discontinuity at standardized biomarker values (SCr/Q < 1 or SCysC/QCC < 1), which lacks a clear biological basis and may influence estimates around this threshold. Schwartz and CKiD equations use a fractional exponent on the height-to-creatinine ratios, which means they are less responsive to changes in this ratio than in a linear model, possibly compressing the upper range of eGFR estimates. The Bedside equation uses the quotient of height and serum creatinine, simplifying a more complex biological reality, but it also leads to implausible high eGFRs as the SCr values approach 0 (however, our lowest value was 0.147 mg/dl), which may nonetheless contribute to the high and widely distributed eGFR estimates observed in younger children in our cohort. Similarly, CAPA uses age multiplied by a negative exponent, meaning the eGFR could technically be infinite, as we approach age 0. Of course, each equation has an intended age range, to avoid these peculiarities, but they may explain some of the larger variations within our results, especially in younger participants.

Equations that tend to yield values within normal/higher ranges (e.g. EKFC, similarly to our study, based on non-CKD cohort) may appear to perform well in this context, not necessarily due to superior accuracy, but because their outputs align with the characteristics of the study population and our selected target eGFR range. Equations based on CKD cohorts often show an underestimation of the GFR in non-CKD populations, which could result in lesser agreement of the eGFR and our 90–120 ml/min/1.73 m² range.

Further, all eGFR equations based on endogenous markers, such as serum creatinine or cystatin C, have a fundamental limitation at high levels of GFR: as GFR increases, the concentrations of these markers decrease, causing the relationship between markers and GFR to flatten, which reduces the sensitivity of the markers to true differences in filtration rate. In this range, the routine analytical imprecision that is inherent in any clinical laboratory setting contributes significantly to variability in eGFR estimates. This constraint cannot be overcome by equation choice alone. It should be considered when interpreting results for healthy children with physiologically high GFR.

A certain degree of circularity in our findings cannot be ruled out, considering our cohort consisted of apparently healthy individuals, therefore this possibly leads to lack of abnormal clinical findings. While we consider the health ascertainment in our cohort to be comprehensive, though we acknowledge that no community-based study can guarantee the absence of all undiagnosed conditions. Therefore, the most significant limitation of our study is the absence of actual measured GFR. Since we did not have measured filtration rates for direct comparison, our research cannot make a definitive statement about their accuracy.

## Conclusion

In our study, we compared eGFRs from a large, apparently healthy cohort of children and adolescents using several estimation equations. While our research cannot pinpoint one formula for estimating kidney function as the most accurate, we were able to show that some formulas led to more implausible results for a pediatric CKD-free cohort than others.

Notably, we observed that simpler equations, such as Schwartz Bedside and CAPA, are more prone to producing extreme outliers and implausible discontinuities between age groups. This tendency suggests that these formulas may be less suitable for estimating kidney function in apparently healthy paediatric, CKD-free populations.

## Supplementary Information

Below is the link to the electronic supplementary material.

Supplementary Material 1

## Data Availability

The data that support the findings of this study are available from LIFE Child-study but restrictions apply to the availability of these data, which were used under license for the current study, and so are not publicly available. Researchers interested in accessing data may contact the study at [https://home.uni-leipzig.de/lifechild/research/].

## Abbreviations

eGFR: Estimated glomerular filtration rate
GFR: Glomerular filtration rate
CKiD: Kidney disease in children
EKFC: European kidney function consortium
SCr: Serum creatinine
CysC: Cystatin C
