# Supplementary material for: Examining commonly used equations for estimating the glomerular filtration rate (GFR) in a healthy cohort of children and adolescents
Source: BMC Nephrol. 2026 Jul 25;27:446. doi: 10.1186/s12882-026-05219-y (PMC13410585; doi:10.1186/s12882-026-05219-y)
Supplement: Supplementary file 1 — Supplementary Material 1 [file 12882_2026_5219_MOESM1_ESM.docx]

Additional File

Examining commonly used equations for estimating the glomerular filtration rate (GFR) in a healthy cohort of children and adolescents

Luise Trenkmann^1^, Niels Ziegelasch^1^, Katalin Dittrich ^2,5^, Anja Willenberg^4^, Wieland Kiess ^1,2^, Mandy Vogel ^1,2^

^1^ LIFE Child Study, University Hospital for Children and Adolescents, Medical Faculty, Leipzig University, Philipp-Rosenthal-Strasse 27, 04103 Leipzig, Germany

^2^ Center of Paediatric Research (CPL), University of Leipzig, 04103 Leipzig, Germany

^3^ Institute for Medical Informatics, Statistics and Epidemiology (IMISE), University of Leipzig, 04107 Leipzig, Germany

^4^ Institute for Laboratory Medicine, Clinical Chemistry and Molecular Diagnostics (ILM), University Hospital Leipzig, 04103 Leipzig, Germany

^5^ Hospital for Children and Adolescents, University of Leipzig, Liebigstraße 20a, 04103 Leipzig, Germany

Additional File S1. Equations used for estimating the GFR

| Equations |  |
| --- | --- |
| Schwartz Bedside (2009)^1^ | $eGFR \left( ml/min/1.73m^{2} \right)=0.413\times\frac{height (m)}{SCr (\frac{mg}{dl})}$ |
| Schwartz (2009)^1^ | $eGFR \left( ml/min/1.73^{2} \right)=39.1\times{\frac{\mathrm{height}\left( m \right)}{\mathrm{Scr}\left( \frac{\mathrm{mg}}{\mathrm{dl}} \right)}}^{0.516} \times{\frac{1.8}{cystatin C (mg/L)}}^{0.294}\times{\frac{30}{BUN (mg/dl)}}^{0.169}\times{[1.099]}^{\mathrm{male}}\times{\frac{\mathrm{height}\left( m \right)}{1.4}}^{0.188}$ |
| CKiD (2012)^2^ | $eGFR \left( ml/min/1.73m^{2} \right)=39.8\times{\frac{\mathrm{height}\left( m \right)}{\mathrm{Scr}\left( \frac{\mathrm{mg}}{\mathrm{dl}} \right)}}^{0.456} \times{\frac{1.8}{cystatin C (mg/L)}}^{0.418}\times{\frac{30}{BUN (mg/dl)}}^{0.079}\times{[1.076]}^{gender}\times{\frac{\mathrm{height}\left( m \right)}{1.4}}^{0.179}$ |
| CAPA (2014)^3^ | $eGFR \left( ml/min/1.73m^{2} \right)=130\times{Cystatin C}^{-1.069}\times{age}^{-0.117}-7$ |
| U25cys (2021)^4^ | $eGFR \left( ml/min/1.73m^{2} \right)=K\times1/{Cystatin C}$ |
| U25scr (2021)^4^ | $eGFR \left( ml/min/1.73m^{2} \right)=K\times{height}/{Scr}$ |
| U25ave (2021)^4^ | $eGFR \left( ml/min/1.73m^{2} \right)=\frac{U25scr+U25cys}{2}$ |
| CKD-EPI (2021)^5^ | $eGFR (ml/min/1.73m^{2})=142\times\min\left( standardized\frac{Scr}{K}, 1 \right)\alpha\times\max\left( standardized\frac{Scr}{K}, 1 \right)-1.200 \times0.9938Age \times1.012 \left[ if female \right]$ |
| EKFCcr^6^ | If SCr/Q <1: $eGFR (ml/min/1.73m^{2})=107.3 \times{(\frac{\mathrm{SCr}}{Q})}^{-0.322}$  If SCr/Q ≥ 1: $eGFR (ml/min/1.73m^{2})=107.3 \times{(\frac{\mathrm{SCr}}{Q})}^{-1.132}$ |
| EKFCcys^7^ | 2 – 40 years, SCysC/Q_CC_ <1: $eGFR (ml/min/1.73m^{2})=107.3 \times{(\frac{\mathrm{SCysC}}{Qcc})}^{-0.322}$ 2 – 40 years, SCysC/Q_CC_ ≥1: $eGFR (ml/min/1.73m^{2})=107.3 \times{(\frac{\mathrm{SCysC}}{Qcc})}^{-1.132}$ |

References

1. Schwartz GJ, Mun[Combining Tilde]oz A, Schneider MF, et al. New Equations to Estimate GFR in Children with CKD. *J Am Soc Nephrol*. 2009;20(3):629-637. doi:10.1681/ASN.2008030287

2. Schwartz GJ, Schneider MF, Maier PS, et al. Improved equations estimating GFR in children with chronic kidney disease using an immunonephelometric determination of cystatin C. *Kidney Int*. 2012;82(4):445-453. doi:10.1038/ki.2012.169

3. Grubb A, Horio M, Hansson LO, et al. Generation of a New Cystatin C–Based Estimating Equation for Glomerular Filtration Rate by Use of 7 Assays Standardized to the International Calibrator. *Clin Chem*. 2014;60(7):974-986. doi:10.1373/clinchem.2013.220707

4. Pierce CB, Muñoz A, Ng DK, Warady BA, Furth SL, Schwartz GJ. Age- and sex-dependent clinical equations to estimate glomerular filtration rates in children and young adults with chronic kidney disease. *Kidney Int*. 2021;99(4):948-956. doi:10.1016/j.kint.2020.10.047

5. Inker LA, Eneanya ND, Coresh J, et al. New Creatinine- and Cystatin C–Based Equations to Estimate GFR without Race. *N Engl J Med*. 2021;385(19):1737-1749. doi:10.1056/NEJMoa2102953

6. Pottel H, Björk J, Courbebaisse M, et al. Development and Validation of a Modified Full Age Spectrum Creatinine-Based Equation to Estimate Glomerular Filtration Rate: A Cross-sectional Analysis of Pooled Data. *Ann Intern Med*. 2021;174(2):183-191. doi:10.7326/M20-4366

7. Pottel H, Björk J, Rule AD, et al. Cystatin C–Based Equation to Estimate GFR without the Inclusion of Race and Sex. *N Engl J Med*. 2023;388(4):333-343. doi:10.1056/NEJMoa2203769

8. De Souza VC, Rabilloud M, Cochat P, et al. Schwartz Formula: Is One k-Coefficient Adequate for All Children? Seguro AC, ed. *PLoS ONE*. 2012;7(12):e53439. doi:10.1371/journal.pone.0053439

Additional File S2. Regarding Participants Health Status:

Health status of participants was determined through a multi-layered, systematic ascertainment process. Each participant underwent a comprehensive anamnestic interview conducted by a pediatrician, covering personal and family medical history, including parents, siblings, and where available, grandparents. A structured review of current and past medications was performed by a pediatrician or a trained and certified study assistant, with medications coded according to the Anatomical Therapeutic Chemical (ATC) Classification System. The Gelbe Heft — the standardized German well-child visit documentation — as well as vaccination records (Impfpässe) were reviewed for each participant. Participants were seen on multiple occasions: three visits during the first year of life, followed by annual follow-up visits, providing a longitudinal and comprehensive picture of each child's health trajectory. Children were excluded from the analysis if a relevant disease or medication (identified by ATC code) was recorded at any point during follow-up.

Additional File S3. Percentage of estimated GFR between 90-135mL/min/1.73m² (N)

| Age | Sex | Percentage of eGFR between 90-135mL/min/1.73 m² (N) | | | | | | | |
| --- | --- | --- | --- | --- | --- | --- | --- | --- | --- |
|  |  | U25ave | U25cys | U25scr | CKD-EPI | Bedside | CAPA | CKiD | Schwartz |
| 2-5 | *female* | 80,14  (448) | 57,24  (352) | 78,14  (454) | 20,93  (117) | 64,20  (373) | 61,63  (379) | 63,47  (106) | 73,65  (123) |
|  | *male* | 83,79  (548) | 48,14  (349) | 82,80  (568) | 20,34  (133) | 69,53  (477) | 67,59  (490) | 83,43  (141) | 94,67  (160) |
| 5-10 | *female* | 58,93  (845) | 48,73  (748) | 63,66  (923) | 70,01  (1004) | 86,76  (1258) | 87,49  (1343) | 56,83  (333) | 70,99  (416) |
|  | *male* | 80,09  (1364) | 59,63  (1084) | 82,66  (1421) | 49,68  (846) | 85,63  (1472) | 86,63  (1575) | 82,47  (555) | 91,98  (619) |
| 10-18 | *female* | 52,42  (1319) | 38,99  (991) | 60,60  (1544) | 93,64  (2356) | 77,08  (1964) | 81,00  (2059) | 57,58  (695) | 72,74  (878) |
|  | *male* | 73,96  (2056) | 48,98  (1374) | 79,36  (2234) | 86,08  (2393) | 70,44  (1983) | 65,45  (1836) | 67,70  (811) | 83,97  (1006) |
| 18+ | *female* | 42,26  (71) | 30,18  (51) | 53,57  (90) | 95,24  (160) | 52,98  (89) | 88,76  (150) | 64,00  (32) | 66,00  (33) |
|  | *male* | 60,87  (84) | 29,79  (42) | 74,65  (106) | 96,38  (133) | 12,68  (18) | 75,89  (107) | 41,18  (7) | 47,06  (8) |

The table shows the percentage of results within the normal eGFR-range, the number of measurements is stated in parentheses. For visual clarity, percentage values are color-coded as follows: green (≥75%), yellow (≥50%), orange (≥25%) and red (<25%).

Additional File S4. Bland-Altman Analysis

This table contains the Bland–Altman analysis comparing the eGFR equations (*Method1, Method2)*, showing mean bias with 95% confidence interval (bias [biasLowerCI:biasUpperCI]), limits of agreement (Upper LOA, Lower LOA), number of observations, and results of regression analysis with fixed slope and intercept.

| Method1 | Method2 | Bias [LowerCI:UpperCI] | Lower LOA | Upper LOA | No. of observations | Regression fixed slope | Regression fixed intercept |
| --- | --- | --- | --- | --- | --- | --- | --- |
| U25ave | U25cys | 5,49 [5,32:5,65] | -11,33 | 22,31 | 10623 | -0,08 | 13 |
| U25ave | U25scr | -5,49 [-5,65:-5,32] | -22,31 | 11,33 | 10623 | -0,37 | 31 |
| U25ave | CKDEPI | -30,67 [-30,88:-30,46] | -52,43 | -8,92 | 10623 | -0,46 | 20 |
| U25ave | Bedside | -14,70 [-14,97:-14,43] | -42,69 | 13,29 | 10623 | -0,67 | 54 |
| U25ave | CAPA | -14,51 [-14,80:-14,22] | -44,67 | 15,65 | 10623 | -0,67 | 55 |
| U25ave | CKiD | 1,86 [1,77:1,96] | -4,29 | 8,01 | 4240 | 0,097 | -7,4 |
| U25ave | schwartz | -2,93 [-3,08:-2,77] | -13,10 | 7,25 | 4240 | -0,02 | -0,92 |
| U25ave | ekfc.cys | -3,05 [-3,23:-2,86] | -21,60 | 15,50 | 9952 | 0,0078 | -3,8 |
| U25ave | ekfc.cr | -3,90 [-4,06:-3,75] | -19,17 | 11,37 | 9952 | -0,062 | 2,2 |
| U25cys | U25scr | -10,97 [-11,30:-10,65] | -44,61 | 22,67 | 10623 | -0,43 | 30 |
| U25cys | CKDEPI | -36,16 [-36,40:-35,92] | -61,06 | -11,26 | 10623 | -0,41 | 8 |
| U25cys | Bedside | -20,19 [-20,59:-19,78] | -61,99 | 21,62 | 10623 | -0,81 | 62 |
| U25cys | CAPA | -20,28 [-20,53:-20,03] | -46,60 | 6,04 | 11060 | -0,55 | 35 |
| U25cys | CKiD | -1,42 [-1,69:-1,15] | -19,00 | 16,17 | 4240 | 0,3 | -29 |
| U25cys | schwartz | -6,21 [-6,55:-5,86] | -28,53 | 16,12 | 4240 | 0,18 | -24 |
| U25cys | ekfc.cys | -7,91 [-8,02:-7,79] | -20,01 | 4,20 | 10350 | 0,1 | -17 |
| U25cys | ekfc.cr | -8,73 [-9,00:-8,46] | -35,49 | 18,03 | 9952 | 0,031 | -12 |
| U25scr | CKDEPI | -25,18 [-25,47-24,90] | -55,05 | 4,68 | 10623 | -0,084 | -16 |
| U25scr | bedside | -9,24 [-9,42:-9,05] | -28,86 | 10,39 | 10787 | -0,27 | 20 |
| U25scr | CAPA | -9,02 [-9,42:-8,62] | -50,15 | 32,10 | 10623 | -0,34 | 27 |
| U25scr | CKiD | 5,14 [4,88:5,41] | -12,21 | 22,49 | 4240 | 0,39 | -33 |
| U25scr | schwartz | 0,35 [0,11:0,59] | -15,24 | 15,95 | 4240 | 0,26 | -26 |
| U25scr | ekfc.cys | 1,78 [1,45:2,10] | -30,44 | 33,99 | 9952 | 0,52 | -50 |
| U25scr | ekfc.cr | 0,95 [0,80:1,11] | -14,71 | 16,62 | 10109 | 0,29 | -28 |
| CKDEPI | bedside | 15,97 [15,69:16,25] | -12,77 | 44,71 | 10623 | -0,23 | 43 |
| CKDEPI | CAPA | 16,16 [15,99:16,33] | -1,72 | 34,04 | 10623 | -0,17 | 37 |
| CKDEPI | CKiD | 32,30 [31,95:32,65] | 9,37 | 55,22 | 4240 | 0,55 | -29 |
| CKDEPI | Schwartz | 27,51 [27,12:27,90] | 2,13 | 52,88 | 4240 | 0,45 | -23 |
| CKDEPI | ekfc.cys | 26,54 [26,32:26,75] | 5,09 | 47,99 | 9952 | 0,46 | -25 |
| CKDEPI | ekfc.cr | 25,68 [25,39:25,98] | -3,37 | 54,73 | 9952 | 0,46 | -26 |
| bedside | CAPA | 0,19 [-0,20:0,58] | -39,97 | 40,35 | 10623 | 0,041 | -4,3 |
| bedside | CKiD | 13,98 [13,59:14,38] | -11,67 | 39,63 | 4240 | 0,65 | -52 |
| bedside | schwartz | 9,19 [8,82:9,57] | -15,37 | 33,75 | 4240 | 0,52 | -45 |
| bedside | ekfc.cys | 10,15 [9,78:10,53] | -27,22 | 47,52 | 9952 | 0,82 | -75 |
| bedside | ekfc.cr | 9,36 [9,11:9,60] | -15,60 | 34,31 | 10109 | 0,56 | -49 |
| CAPA | CKiD | 17,87 [17,35:18,39] | -15,84 | 51,57 | 4240 | 0,85 | -70 |
| CAPA | schwartz | 13,08 [12,51:13,65] | -24,04 | 50,20 | 4240 | 0,8 | -71 |
| CAPA | ekfc.cys | 10,40 [10,18:10,62] | -11,71 | 32,52 | 10350 | 0,6 | -52 |
| CAPA | ekfc.cr | 9,30 [8,91:9,69] | -29,56 | 48,15 | 9952 | 0,77 | -71 |
| CKiD | schwartz | -4,79 [-4,88:-4,70] | -10,85 | 1,27 | 4240 | -0,12 | 6,6 |
| CKiD | ekfc.cys | -5,74 [-6,02:-5,47] | -23,55 | 12,06 | 4067 | -0,12 | 5,6 |
| CKiD | ekfc.cr | -4,73 [-4,98:-4,48] | -20,77 | 11,31 | 4067 | -0,13 | 8,1 |
| schwartz | ekfc.cys | -1,05 [-1,41:-0,70] | -23,65 | 21,54 | 4067 | 0,024 | -3,4 |
| schwartz | ekfc.cr | -0,04 [-0,29:0,21] | -15,95 | 15,88 | 4067 | -0,0011 | 0,068 |
| ekfc.cys | ekfc.cr | -0,85 [-1,13:-0,58] | -27,97 | 26,26 | 9952 | -0,098 | 8,9 |


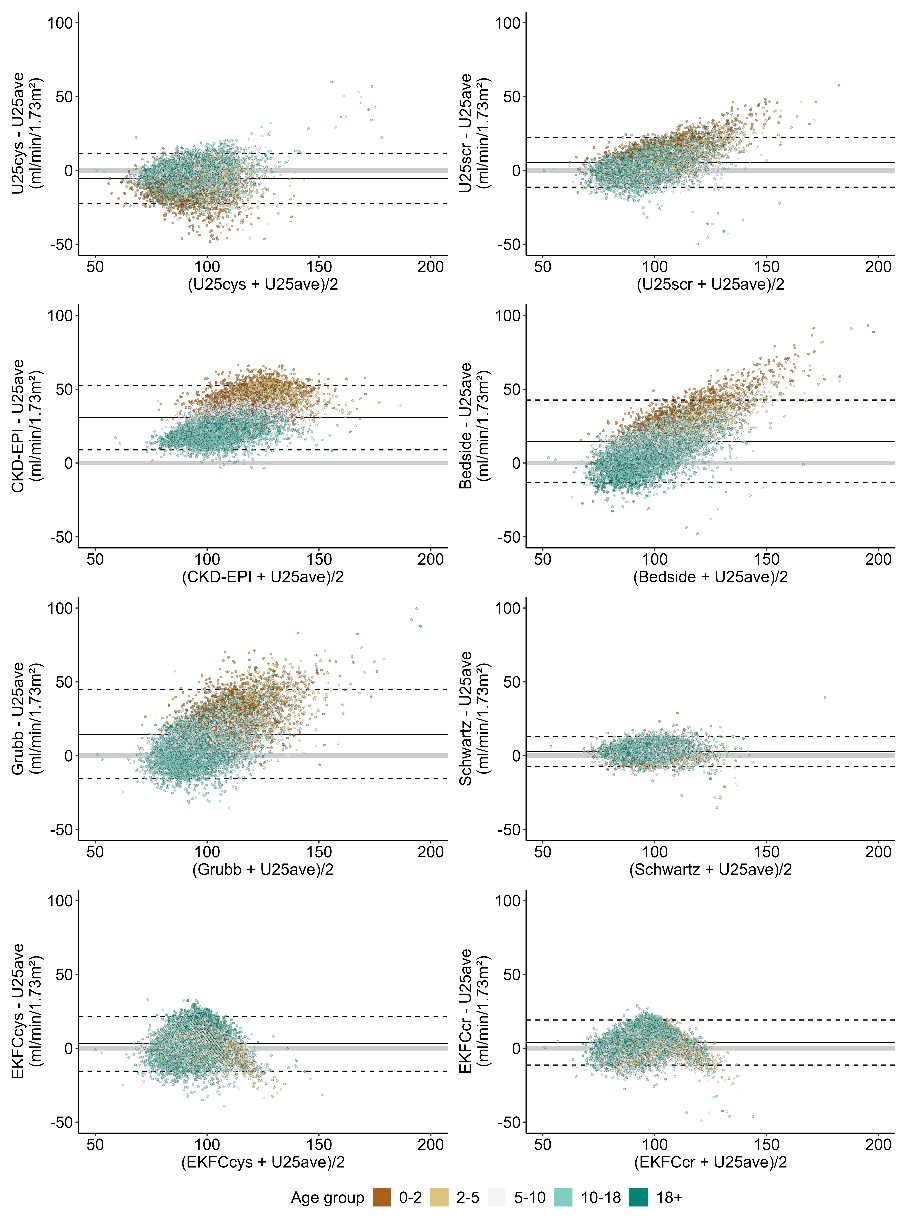
 Additional File S5. Bland-Altman-Plots plotting the difference between the equations against their averages, grouped by age
The solid black horizontal lines indicate mean bias, the solid light-grey line shows zero bias, while the dashed lines represent ±1.96 standard deviations (limits of agreement).


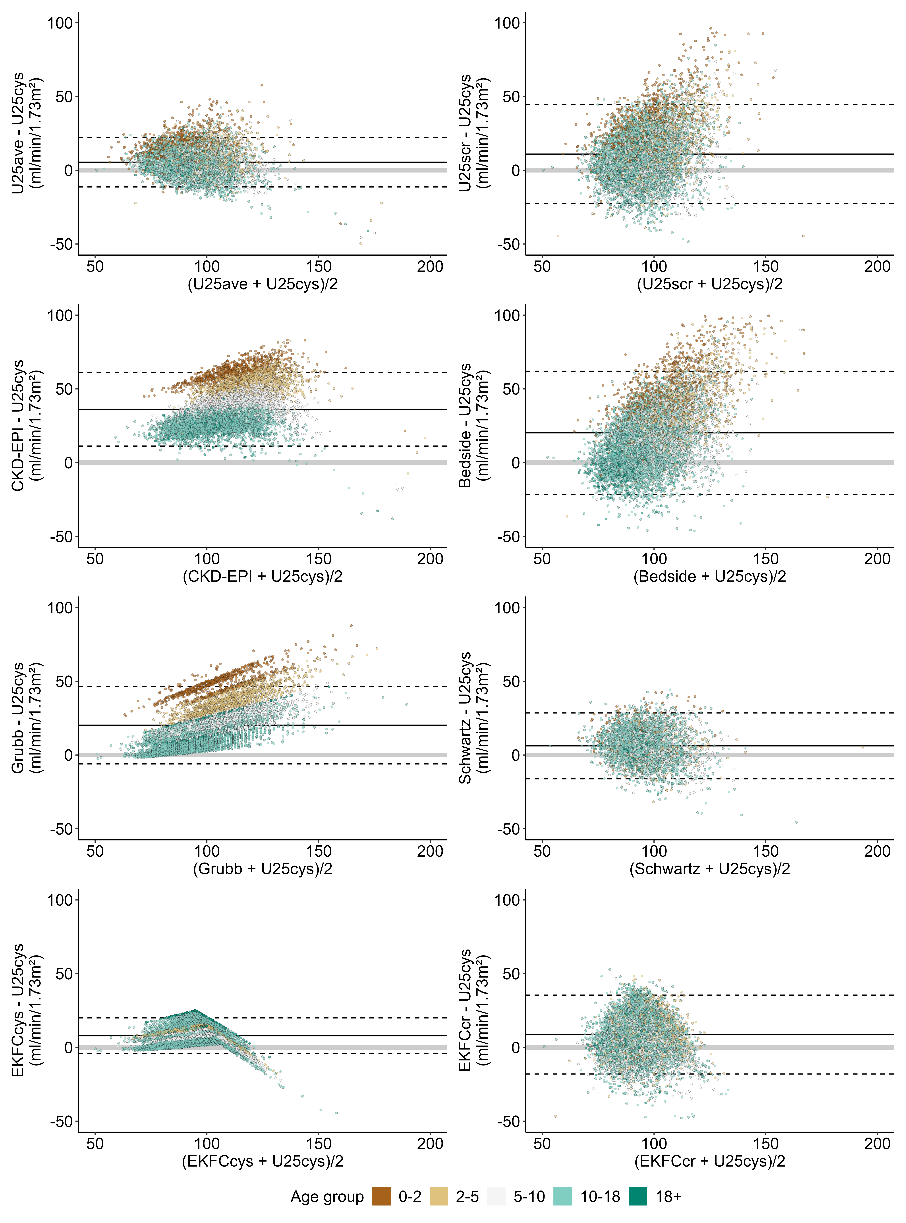
Additional File S6. Bland-Altman-Plots plotting the difference between the equations against their averages, grouped by age
The solid black horizontal lines indicate mean bias, the solid light-grey line shows zero bias, while the dashed lines represent ±1.96 standard deviations (limits of agreement).

Additional File S7. Bland-Altman-Plots plotting the difference between the equations against their averages, grouped by age
The solid black horizontal lines indicate mean bias, the solid light-grey line shows zero bias, while the dashed lines represent ±1.96 standard
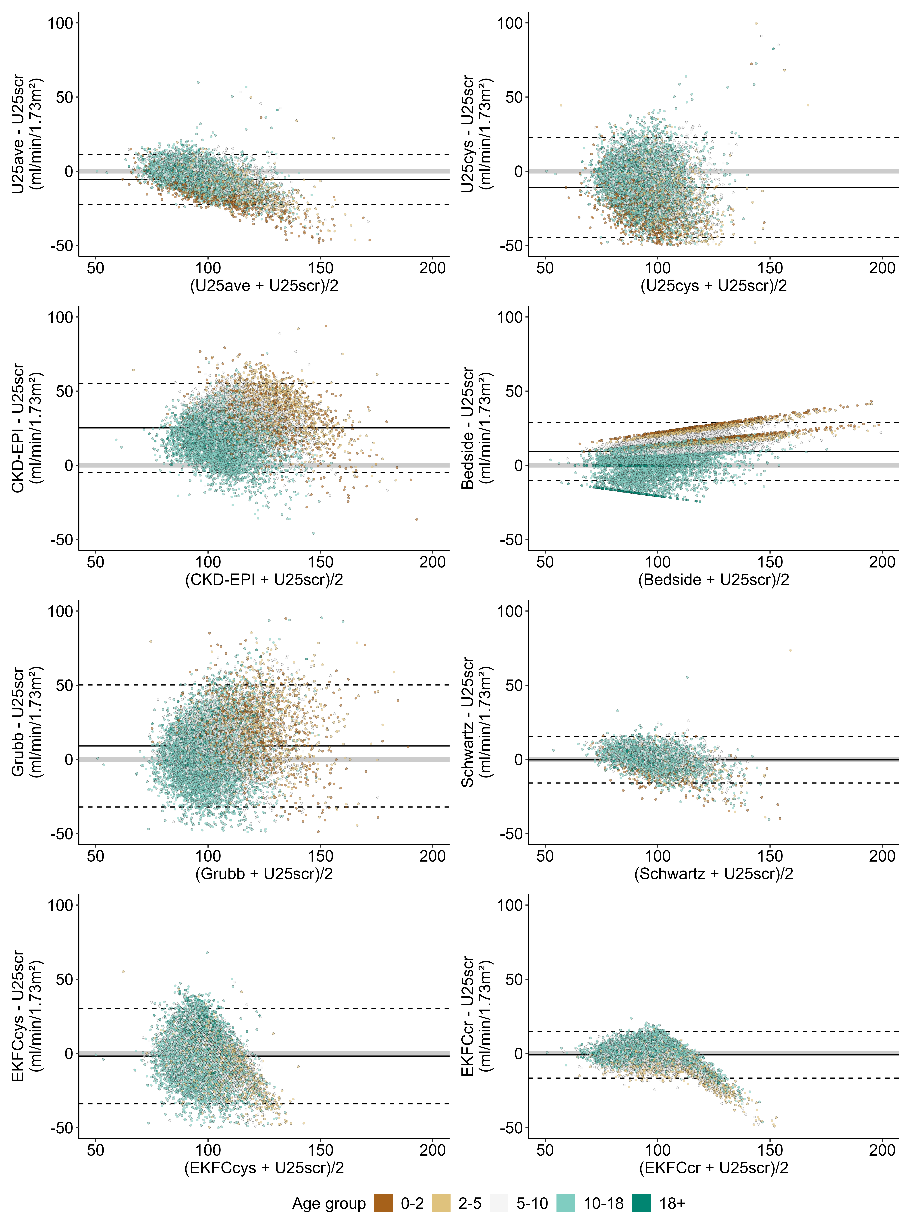
deviations (limits of agreement).


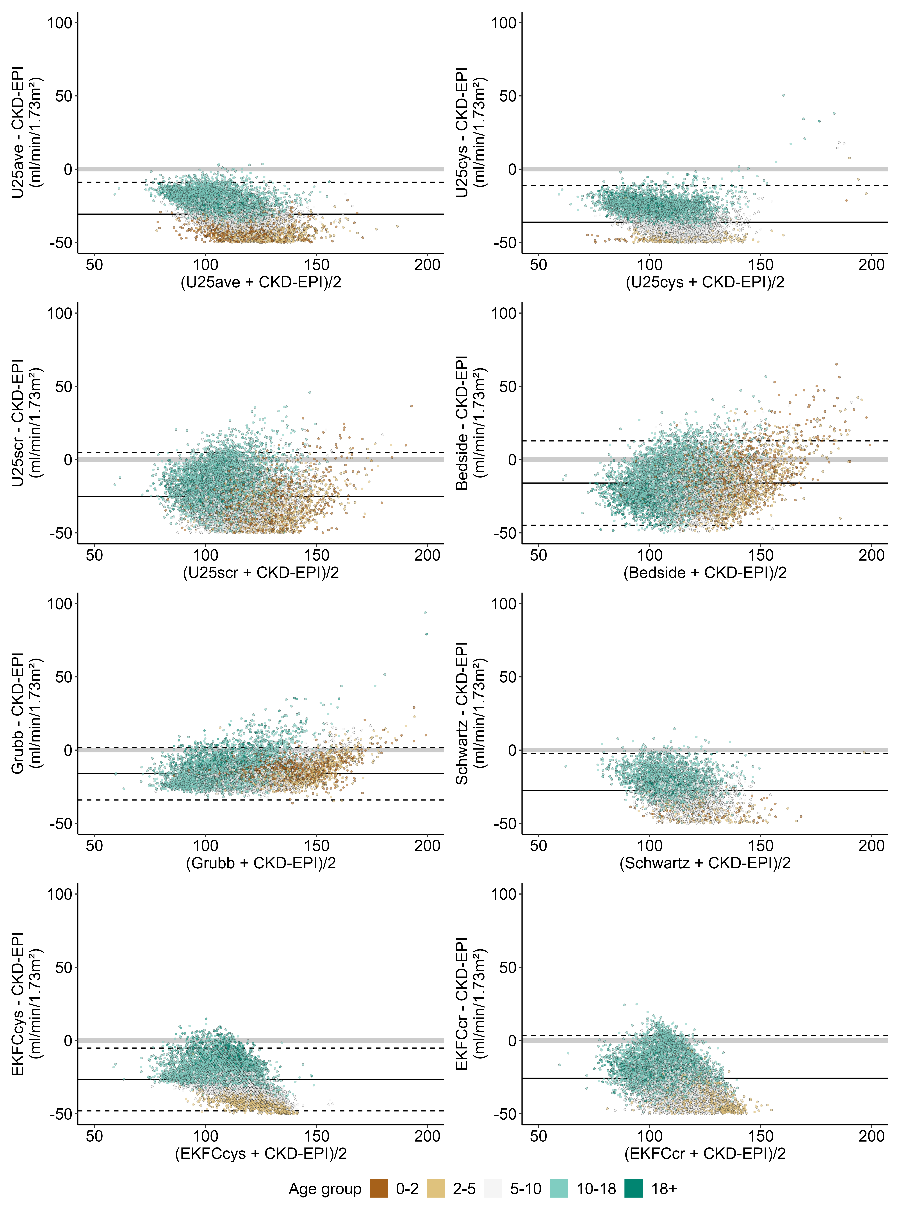
Additional File S8. Bland-Altman-Plots plotting the difference between the equations against their averages, grouped by age
The solid black horizontal lines indicate mean bias, the solid light-grey line shows zero bias, while the dashed lines represent ±1.96 standard deviations (limits of agreement).


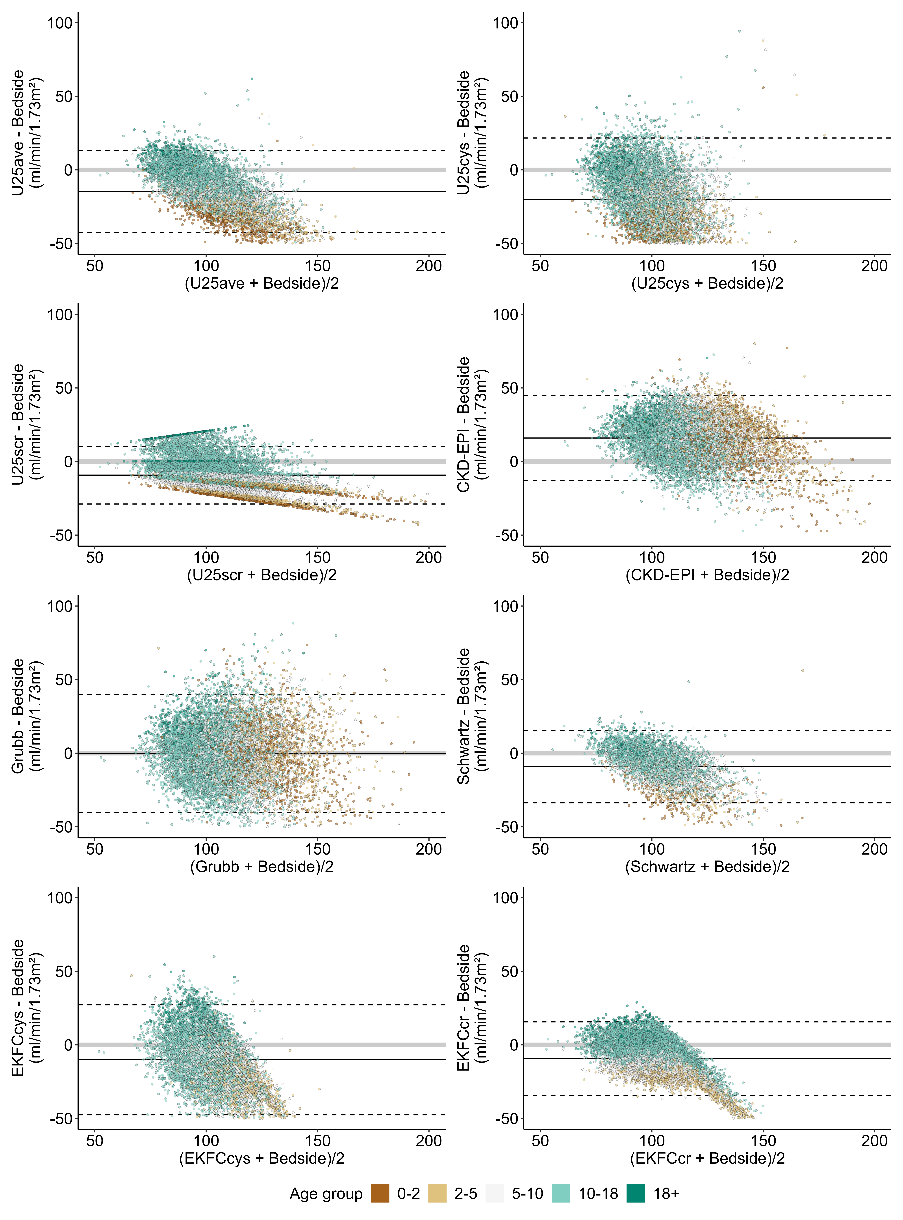
Additional File S9. Bland-Altman-Plots plotting the difference between the equations against their averages, grouped by age
The solid black horizontal lines indicate mean bias, the solid light-grey line shows zero bias, while the dashed lines represent ±1.96 standard deviations (limits of agreement).


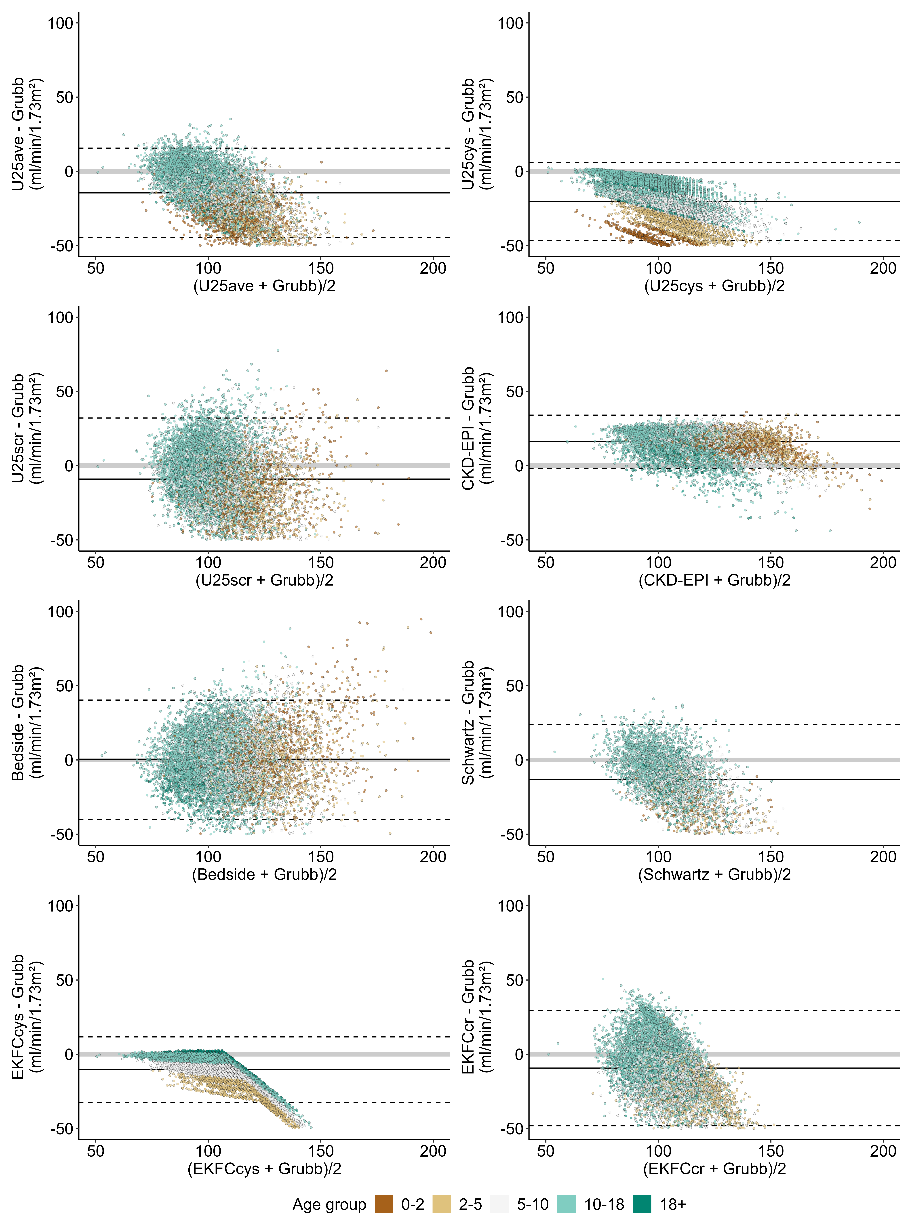
Additional File S10. Bland-Altman-Plots plotting the difference between the equations against their averages, grouped by age
The solid black horizontal lines indicate mean bias, the solid light-grey line shows zero bias, while the dashed lines represent ±1.96 standard deviations (limits of agreement).


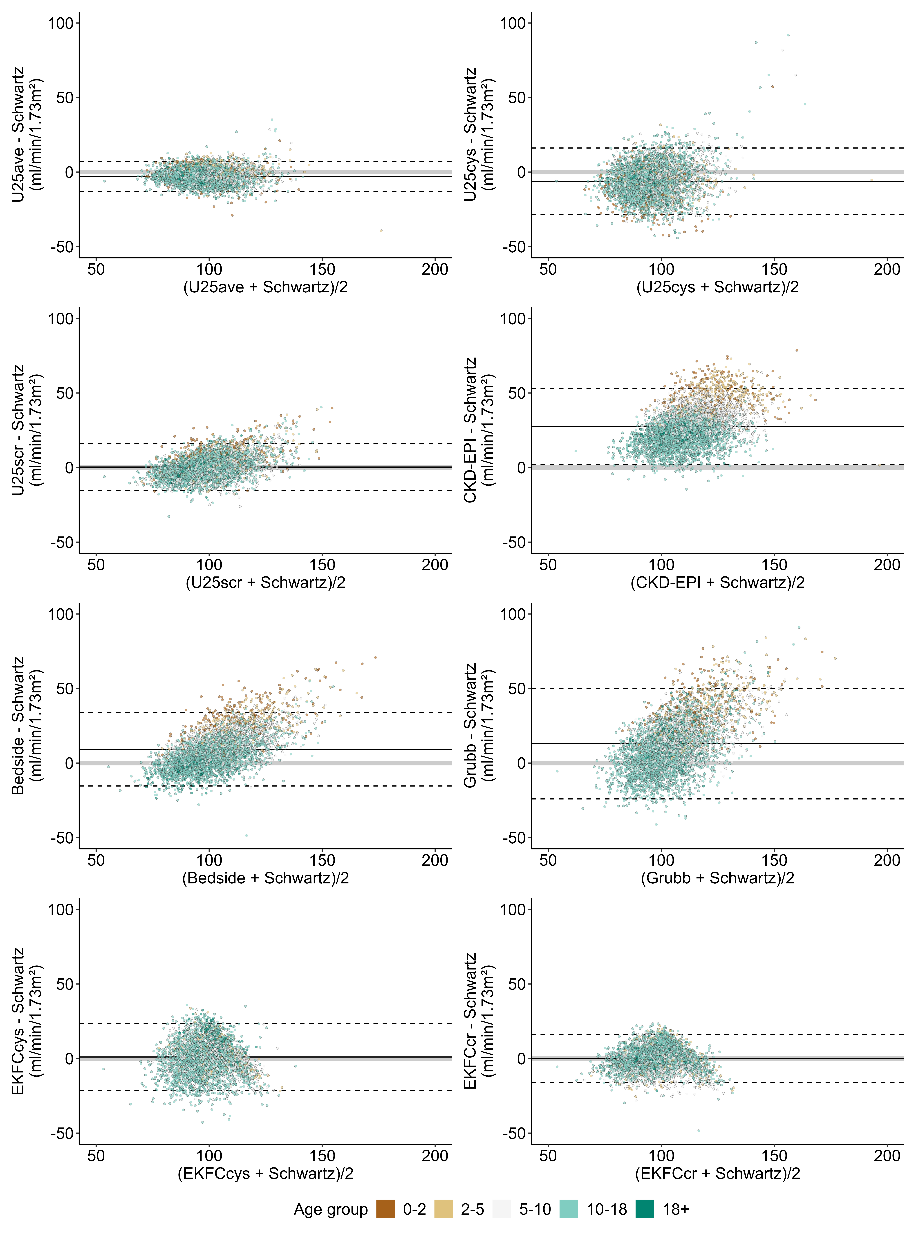
Additional File S11. Bland-Altman-Plots plotting the difference between the equations against their averages, grouped by age
The solid black horizontal lines indicate mean bias, the solid light-grey line shows zero bias, while the dashed lines represent ±1.96 standard deviations (limits of agreement).


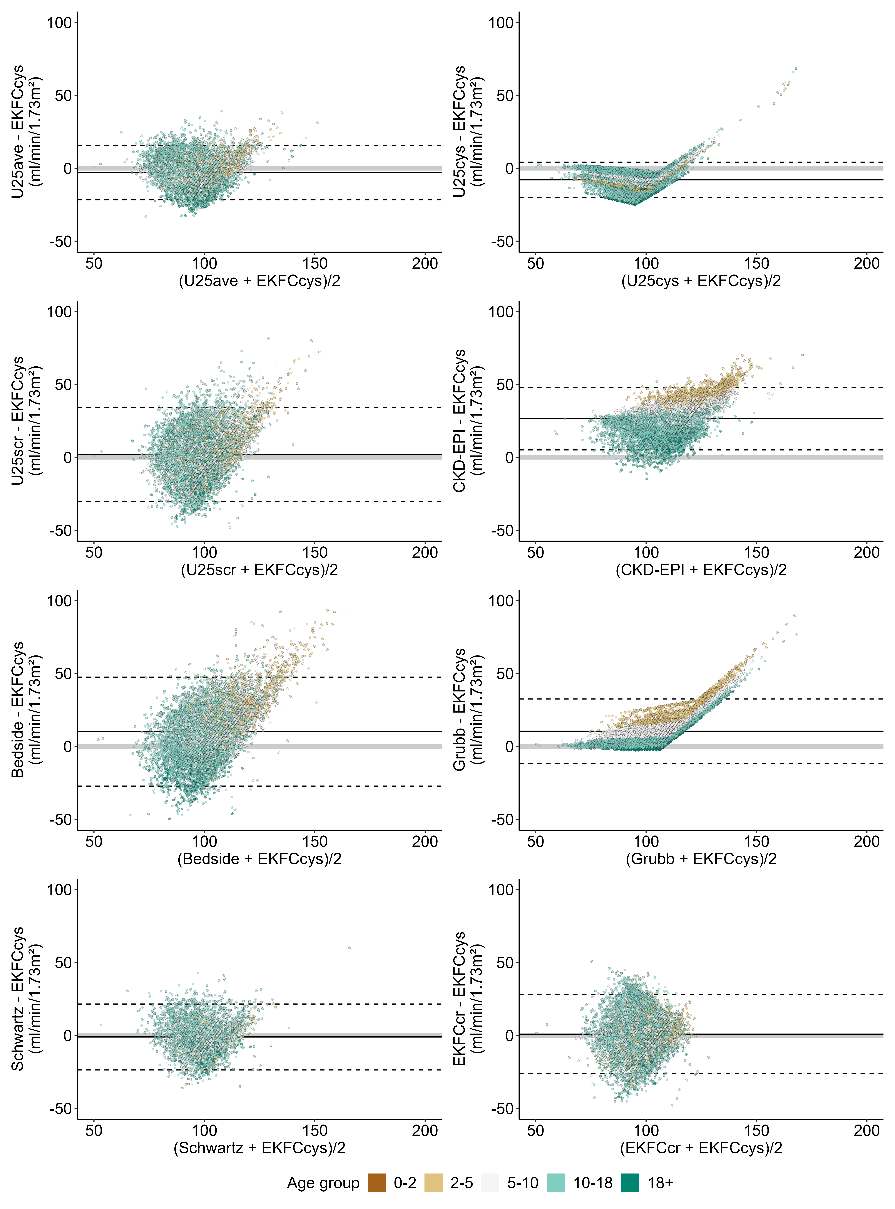
Additional File S12. Bland-Altman-Plots plotting the difference between the equations against their averages, grouped by age
The solid black horizontal lines indicate mean bias, the solid light-grey line shows zero bias, while the dashed lines represent ±1.96 standard deviations (limits of agreement).


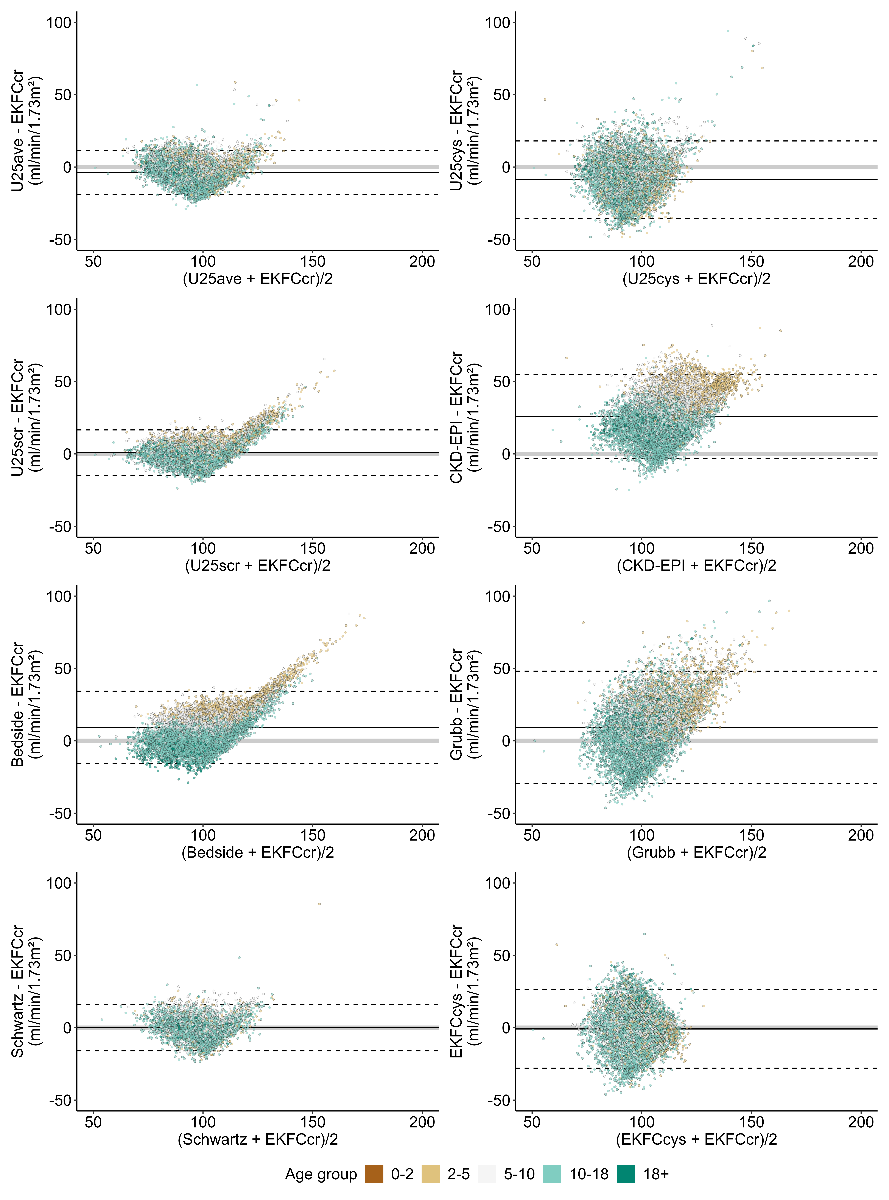
Additional File S13. Bland-Altman-Plots plotting the difference between the equations against their averages, grouped by age
The solid black horizontal lines indicate mean bias, the solid light-grey line shows zero bias, while the dashed lines represent ±1.96 standard deviations (limits of agreement).

Additional File S14. Percentage of estimated GFR between 90-120ml/min/1.73m² (N) grouped by age (years)

The table shows the percentage of results within the normal eGFR-range, the number of measurements is stated in parentheses. For visual clarity, percentage values are color-coded as follows: green (≥75%), yellow (≥50%), orange (≥25%) and red (<25%).

| Age | Sex | Percentage of eGFR between 90-120ml/min/1.73 m² (N) | | | | | | | | | | |
| --- | --- | --- | --- | --- | --- | --- | --- | --- | --- | --- | --- | --- |
|  |  | U25ave | U25cys | U25scr | CKD-EPI | Bedside | Schwartz-Lyon^8^ | CAPA | CKiD | Schwartz | EKFCcys | EKFCcr |
| 2-12 | *female* | 61.21  (1665) | 47.40  (1369) | 62.34  (1725) | 29.67  (807) | 60.32  (1669) | 66.93  (1852) | 59.76  (1726) | 57.18  (629) | 70.82  (779) | 80.62  (2321) | 77.66  (2142) |
|  | *male* | 76.71  (2454) | 56.28  (1910) | 70.33  (2287) | 14.16  (453) | 59.27  (1934) | 67.80  (2205) | 60.37  (2049) | 78.90  (965) | 83.89  (1026) | 81.37  (2751) | 80.75  (2617) |
| 12-17 | *female* | 50.59  (820) | 38.50  (628) | 56.36  (926) | 62.18  (1008) | 65.98  (1084) | 45.77  (752) | 67.87  (1107) | 57.47  (458) | 70.64  (563) | 82.22  (1341) | 69.20  (1137) |
|  | *male* | 69.51  (1238) | 42.86  (771) | 69.98  (1266) | 66.48  (1184) | 58.60  (1060) | 56.66  (1025) | 53.86  (969) | 62.06  (481) | 77.16  (598) | 50.92  (916) | 79.71  (1442) |
| 17-25 | *female* | 42.03  (145) | 31.05  (109) | 49.13  (170) | 66.67  (230) | 51.45  (178) | 21.68  (75) | 70.37  (247) | 57.26  (67) | 61.54  (72) | 94.59  (332) | 63.01  (218) |
|  | *male* | 62.75  (192) | 30.42  (94) | 71.47  (223) | 72.55  (222) | 17.63  (55) | 17.63  (55) | 66.99  (207) | 45.16  (28) | 59.68  (37) | 72.49  (224) | 62.18  (194) |

Additional File S15. Boxplot of eGFRs grouped by age and equation


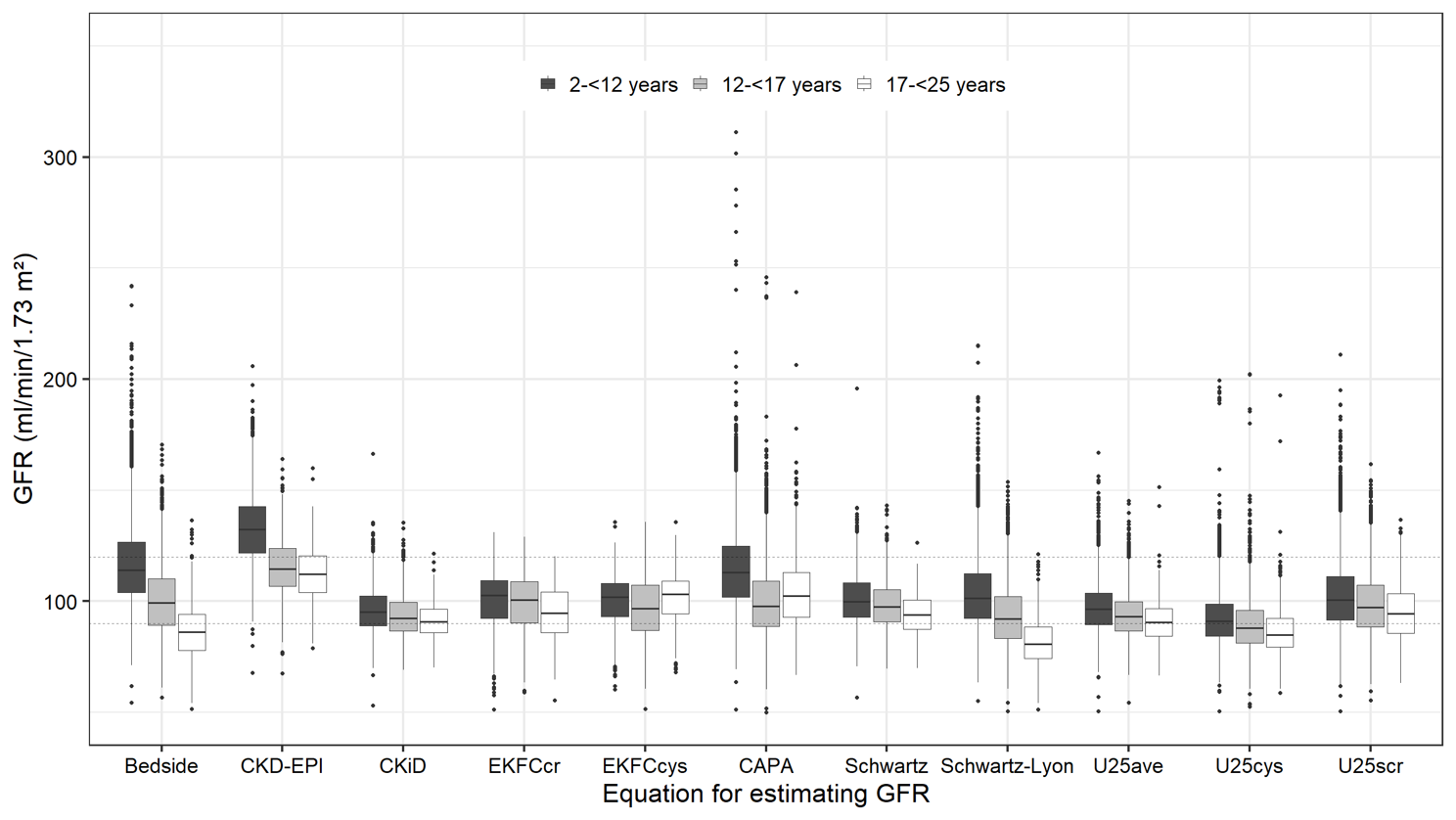
This figure contains two panel boxplots showing GFR estimates (ml/min/1.73 m²) from different GFR estimating equations, stratified by age group. The dotted line represents the upper and lower cutoffs for the GFR in apparently healthy pediatric older than 2 years of age (90-120 ml/min/1.73m²)

Additional File S16. Median serum creatinine by year of age and sex

This table shows the median serum creatinine by year of age and sex in µmol/L and the 95% Confidence Interval.

| **Predicted Median by Age and Sex** | | |
| --- | --- | --- |
| Median (95% Confidence Interval) | | |
| **Age** | **Median (95% CI)***1* | |
|  | **Male** | **Female** |
| **0.5** | 22.10 (21.92-22.29) | 21.78 (21.58-21.98) |
| **1.0** | 21.92 (21.74-22.10) | 21.47 (21.28-21.65) |
| **2.0** | 23.16 (22.99-23.33) | 22.22 (22.05-22.40) |
| **3.0** | 26.86 (26.68-27.04) | 26.05 (25.86-26.24) |
| **4.0** | 30.93 (30.74-31.12) | 30.09 (29.90-30.29) |
| **5.0** | 34.05 (33.86-34.24) | 33.69 (33.49-33.89) |
| **6.0** | 37.24 (37.06-37.42) | 37.27 (37.08-37.47) |
| **7.0** | 40.28 (40.10-40.46) | 40.45 (40.26-40.64) |
| **8.0** | 43.56 (43.38-43.73) | 43.32 (43.13-43.50) |
| **9.0** | 46.15 (45.98-46.32) | 45.74 (45.56-45.92) |
| **10.0** | 48.45 (48.28-48.63) | 48.23 (48.05-48.41) |
| **11.0** | 50.63 (50.45-50.81) | 49.65 (49.46-49.84) |
| **12.0** | 52.85 (52.65-53.05) | 51.81 (51.60-52.02) |
| **13.0** | 56.76 (56.53-56.99) | 55.58 (55.34-55.82) |
| **14.0** | 63.01 (62.73-63.29) | 59.87 (59.58-60.17) |
| **15.0** | 70.81 (70.45-71.17) | 63.57 (63.22-63.91) |
| **16.0** | 76.66 (76.23-77.09) | 65.06 (64.66-65.46) |
| **17.0** | 80.25 (79.75-80.75) | 66.49 (66.04-66.95) |
| **18.0** | 83.89 (83.31-84.46) | 66.58 (66.08-67.08) |
| *1* CI = Confidence Interval. Estimates derived from model predictions. | | |
